# Does a decision aid improve informed choice in mammography screening? Results from a randomised controlled trial

**DOI:** 10.1371/journal.pone.0189148

**Published:** 2017-12-13

**Authors:** Maren Reder, Petra Kolip

**Affiliations:** 1 Department of Prevention and Health Promotion, School of Public Health, Bielefeld University, Bielefeld, Germany; 2 Institute of Psychology, University of Hildesheim, Hildesheim, Germany; TNO, NETHERLANDS

## Abstract

**Background:**

Decision aids can support informed choice in mammography screening, but for the German mammography screening programme no systematically evaluated decision aid exists to date. We developed a decision aid for women invited to this programme for the first time based on the criteria of the International Patient Decision Aids Standards Collaboration.

**Objective:**

To determine whether a decision aid increases informed choice about mammography screening programme participation.

**Methods:**

A representative sample of 7,400 women aged 50 was drawn from registration offices in Westphalia-Lippe, Germany. Women were randomised to receive usual care (i.e., the standard information brochure sent with the programme’s invitation letter) or the decision aid. Data were collected online at baseline, post-intervention, and 3 months follow-up. The primary outcome was informed choice. Secondary outcomes were the constituents of informed choice (knowledge, attitude, intention/uptake), decisional conflict, decision regret, and decision stage. Outcomes were analysed using latent structural equation models and *χ*^2^-tests.

**Results:**

1,206 women participated (response rate of 16.3%). The decision aid increased informed choice. Women in the control group had lower odds to make an informed choice at post-intervention (OR 0.26, 95% CI 0.18-0.37) and at follow-up (OR 0.66, 95% CI 0.46-0.94); informed choices remained constant at 30%. This was also reflected in lower knowledge and more decisional conflict. Post-intervention, the uptake intention was higher in the control group, whereas the uptake rate at follow-up was similar. Women in the control group had a more positive attitude at follow-up than women receiving the decision aid. Decision regret and decision stage were not influenced by the intervention.

**Conclusion:**

This paper describes the first systematic evaluation of a newly developed decision aid for the German mammography screening programme in a randomised controlled trial. Our decision aid proved to be an effective tool to enhance the rate of informed choice and was made accessible to the public.

**Trial registration:**

German Clinical Trials Register DRKS00005176.

## Introduction

Women aged 50 to 69 are invited to participate in the German mammography screening programme (MSP). However, informed choice is only achieved in a small proportion of decisions made about participation in the MSP [1]. This uninformed compliance [2] is a major public health problem, mainly because it is unclear whether mammography screening is beneficial [3]. Many women expect unrealistic benefits of this screening [4]. A decision is classified as informed, if the decision maker has good knowledge about the options, her/his attitude is congruent with the decision, and she/he then implements this decision [5]. To enable women invited to the MSP to such an informed choice, they need to be informed about the existing benefits and harms including their probability and to be supported in clarifying the meanings of those benefits and harms for themselves [6]. Importantly, no correct course of action can be determined [7]—only a personally preferred course of action.

Decision aids (DAs) are an effective way to support informed choices: they improve knowledge about options, increase active engagement in decision making, lead to a higher proportion of choices being in congruence with the decision maker‘s values, and lower decisional conflict due to feeling uninformed or unclear about ones values [8]. A typical DA presents risk information both numerically and graphically and includes a type of values clarification exercise. The International Patient Decision Aid Standards Collaboration (IPDAS) [9] developed very comprehensive and widely used standards for DAs. In the context of mammography screening, DAs have the potential to increase the proportion of women making informed choices [10].

Unfortunately, the magnitude of information materials for all kinds of health decisions is contrasted by a paucity of high-quality decision aids [11]. Lenz et al., taking an inventory of German language DAs, found 12 DAs that had been evaluated in a RCT [11] but none of these was on mammography screening. Overall, two types of information materials on the MSP are available: (1) materials published by the Kooperationsgemeinschaft Mammographie which also offers the screening itself; and (2) materials published by health insurances. None of the materials available for the German MSP at the time our study were sufficiently in line with IPDAS criteria [9] or systematically evaluated in a RCT. We therefore developed an evidence-based online interactive decision aid for the German MSP. Recently and after the end of our data collection, a printed DA has been developed by the IQWiQ [12] but it has not been systematically evaluated in a RCT yet.

Internationally, DAs on mammography screening have been systematically evaluated. The Cochrane Review [8] reports two DAs on mammography screening [10, 13] (see DAs 1 and 2 described below). Completively, the Inventory of Decision Aids of the Ottawa Hospital Research Institute [14] yields 5 DAs (other than our DA) on mammography screening: (1) a DA for 40-year old women [13], (2) a DA for 70-year old women [10], (3) a DA for women ages 40-49 for deciding screening start age and interval [15], (4) a DA for women with dense breasts [16], and (5) a DA with the options of screening start at 40 or 50 [17]. None of these DAs is targeted at an average risk population ages 50-69 and three did not present not having a mammography as an option but instead compared different starting ages, screening intervals or additional screening tests.

The DA for women aged 40 improved knowledge and increased the proportion of women who had made a choice but did not affect the proportion of informed choices [13]. The DA for women aged 70 increased knowledge and proportion of informed choices [10]. In a RCT comparing a DA for women aged 50 with information on overdetection to a DA without such information [18], more women in the intervention group had adequate knowledge, fewer had a positive attitude, and fewer intended to be screened. In a pre-post study with 75 women ages 40 to 49 [19], the DA reduced decisional conflict and had no effect on intention. Therefore, we expect positive effects of our DA on informed choice, knowledge and decisional conflict.

### Study objectives

The present study aimed to assess for the first time in Germany the effect of an interactive online DA on informed choice in a RCT with 3 months follow-up. This RCT compared women receiving a DA additional to usual care (i.e., the brochure of the MSP [20]) to women only receiving usual care. The DA included both additional information (all cause mortality) as well as a different presentation (crowd figure pictograms and values clarification exercise). The primary objective was to assess whether the DA increases the proportion of women making an informed choice. The secondary objectives were to evaluate whether the DA (1) increases knowledge about the MSP, (2) changes attitudes on the MSP, (3) changes participation intentions, (4) reduces decisional conflict, and (5) reduces decision regret.

## Methods

This study was conducted in Wesphalia-Lippe, Germany. In our non-blinded two-armed RCT (German Clinical Trials Register DRKS00005176), the participating women were randomised to receive either the DA (intervention group) or usual care (control group). The usual care for women aged 50 in Germany involved, at the time of the study, an invitation to the MSP accompanied by an information brochure (see [20]). This brochure contained written and numerical information about the MSP. Both study groups received these standard materials; the intervention group additionally received the DA. The online assessments were conducted at baseline (T1), post-intervention (T2, two weeks after T1), and 3 months follow-up (T3).

The Ethics Commission of the Medical Association Westphalia-Lippe and the Medical Faculty of the University of Münster approved our study protocol. It was originally planned that women would receive the baseline questionnaire and the DA as well as the post questionnaire in one session. The baseline assessment was moved to two weeks before the intervention and post questionnaire to keep the time needed to work through the DA and respond to the questionnaire to an acceptable level.

Our study invitation contained information about the content, purpose, and procedure of the study including the information that our trial was conducted independent of the MSP. Once written informed consent was obtained, the participating women were randomised to the intervention or control group by the researchers through an allocation sequence generated by a random number generator (Random.org). Women were only informed about their study group at the second assessment, when they either received the DA or only the questionnaire.

The invitation to the study was sent by post 3 weeks before the estimated arrival of the MSP invitation (for more details, see the study protocol [6]). Three weeks after the postal invitation, women consenting to study participation and providing their e-mail address received the link to the baseline questionnaire (T1). Women were e-mailed the link to the second assessment (T2) 2 weeks after the baseline assessment. At T2, women in the intervention group received a link to the DA and the second assessment whereas women in the control group received a link only to the second assessment. The link to the third assessment (T3) was e-mailed to the women 3 months after T2. The screening appointment was assumed to have passed at this time. A reminder was e-mailed 10 days after each survey. Participating women received all 3 e-mails irrespective of their response to the previous questionnaire. Data were collected between April and November 2014. All questionnaires were based on the questionnaire of the study ‘Informed Choice of German and Turkish Women for Participation in the MSP (InEMa)’ [21]. Modifications were made to make it suitable for evaluating an intervention and to be used Online (for the original questionnaire see [1]). All assessments were linked to each other through a self-generated code.

### Participants and recruitment

The sample of 7,400 women was randomly drawn from registration offices in Westphalia-Lippe, North Rhine-Westphalia, Germany. Women were eligible for the trial if they were aged 50 (birth months of March to May 1964). Women with a potential Turkish migration background (according to a name algorithm [22]) were assigned to the InEMa study and accordingly, our sample comprised only women without Turkish migration background.

### Decision aid

The intervention group received an online DA which was designed to comply with IPDAS criteria (see the BARMER website where our DA (the DA is in German) was made available after the end of our study (https://www.barmer.de/gesundheit/praevention/krebspraevention/krebsfrueherkennung/mammographie-13876) and the Decision Aid Library Inventory where it was registered (https://decisionaid.ohri.ca/AZsumm.php?ID=1673)).

Our Online DA consisted of a static information part and an interactive part (see study protocol for a more detailed description [6]). Mathieu et al.’s DA [13] provided the basis for developing the structure of our DA. To meet IPDAS criteria [23], our DA presented the decision options of participation or non-participation in the MSP in their relevant context.

In the information part, the chance of each outcome was expressed as event rate per 200 women screened every 2 years for 20 years using absolute numbers accompanied by crowd figure pictograms (see additional file of the protocol [6]). The advantages and disadvantages of the MSP and their probabilities were described. This was compared to the option of no screening. The information women receive with the brochure in the MSP invitation [20] was included in the information part of our DA (see quantitative information in Table 1).

**Table 1:** Comparison between decision aid and information brochure.

	Information brochure	Decision aid
General description	Paper based booklet / PDF, 12 pages	Online decision aid, information part and interactive part with 3 steps (assigning the information items to categories, rating the importance of each information item, making a choice)
Visual aspects	Short texts, no graphics or pictures, use of arrows as bullet points, questions as headings	Short texts, bullet points, questions as headings, coloured text boxes, crowd figure pictograms, rating scales, graphical summary of personal responses, downloadable personal PDF at the end
Key factual content	General information about the MSP, quality of the MSP, breast cancer and its risk factors, screening procedure, interval cancers and symptoms, follow-up diagnostics, advantages and disadvantages of the MSP	Target group of the DA, breast cancer mortality, overall mortality, true positives, false positives, interval cancers, overdiagnoses, screening procedure, symptoms of breast cancer
Quantitative information	Number of: positive and negative screening results follow-up diagnostics and biopsies breast cancer diagnoses interval cancers breast cancer deaths with mammography screening additional deaths without mammography screening overdiagnoses	Number of: breast cancer deaths with and without mammography screening all-cause deaths with and without mammography screening negative screening results positive screening results/ follow-up diagnostics breast cancer diagnoses interval cancers overdiagnoses
Presentation of quantitative information	Absolute numbers presented in text (200 women with biannual mammography screening over 20 years)	Absolute numbers supported by 3 crowd figure pictograms consisting of 200 female pictograms (200 women over 20 years): (1) breast cancer mortality with biannual mammography screening, (2) breast cancer mortality without mammography screening, (3) false positives, breast cancer diagnoses, and interval cancers with biannual mammography screening
Values clarification exercise	None	Interactive personal work sheet, evaluating information as in favour of or against mammography screening, evaluating importance of information, making a decision about participation in the MSP, input window for remaining questions, downloadable PDF summarising information and personal responses

The interactive part of the DA summarised the main points of the information part and encouraged engagement with the information. It consisted of three steps. (1) The women assigned the information items to the categories ‘in favour of mammography screening’, ‘neither for nor against such screening’, or ‘against the screening’. (2) They rated the importance of each information item for the decision. (3) They made a choice. At the end, the participants received a tailored summary based on their responses.

The content, design, and layout of the DA was informed by qualitative interviews with women and by consulting experts for women‘s health and prevention (see the study protocol [6]). The findings of pre-testing the DA resulted in additionally including the likelihood of all cause mortality. Information on all cause mortality was not part of the brochure and the first draft of our DA. Since both experts and women in the qualitative pre-test, thought information on this desirable, we included this information in our DA. As a result, the information content of the DA differed from that of the brochure.

### Outcome measures

#### Primary outcome measure

According to the three-dimensional classification framework of Marteau et al. [5], the following dimensions were assessed and the continuous scales were then dichotomously coded: (1) knowledge about the screening, (2) attitude towards the screening, and (3) intention/uptake (depending on measurement point). A choice to take part in the MSP was considered to be informed, if a woman had adequate knowledge, positive attitude and positive intention/uptake [5]. A choice not to participate in the MSP was considered to be informed, if a woman had adequate knowledge, negative attitude and no intention/uptake [5]. Therefore, a dichotomous primary outcome resulted (informed/uninformed). Informed choice is thus dependent on both the dimensions used as well as the cut-points employed to dichotomise the continuous dimensions knowledge and attitude.

#### Secondary outcome measures

We used a similar approach to assess knowledge as in a previous study on screening decisions [1] using seven multiple choice items: (1) target group of the MSP, (2) number of women receiving a positive result, (3) whether a positive screening result equals a diagnosis, (4) existence of false negatives, (5) number of diagnoses in screened vs unscreened populations, (6) number of breast cancer deaths in screened vs unscreened populations, and (7) existence of overtreatment. Questions 1 and 3 to 7 assessed conceptual knowledge. Only Question 2 assessed numerical knowledge with four value ranges as response options. This numerical information was considered to be especially important as the number of women receiving a positive mammography screening result (as opposed to a negative screening result) is the most proximal screening outcome. Each correct answer was scored 1 point; items were then summed to calculate the knowledge index (range 0 to 7). To calculate informed choice, the knowledge index was dichotomised. We used a marking scheme for the knowledge index that is similar to previous research [10, 24] and decided a priori that a mark of 50% or above (score > 3) would be considered as adequate knowledge (see study protocol for a more detailed description of all outcome measures [6]).

Attitude was measured according to the reasoned action approach of Fishbein and Ajzen [25]. The four items were adapted from Marteau et al. [5] for use with mammography screening and rated on a five-point scale. To calculate informed choice, the scale ranging from -8 to +8 was dichotomised: a scale score of ≥ 0 represented a positive attitude.

Intention to participate in the MSP and self-reported uptake were each measured using one item: intention to participate in the MSP in the next 3 months (yes/no/undecided) and uptake of the MSP in the last 3 months (MSP/opportunistic screening/none). To calculate informed choice, intention was dichotomised as ‘participation in the MSP in the next 3 months’ and ‘no participation in the MSP in the next 3 months’ and undecided women were excluded. In addition, women who reported at T2 to want to participate in opportunistic screening were excluded. Consequently, only those were categorised as women with a positive intention, who intended to participate in the MSP in the next 3 months (this category did not include women intending to participate in opportunistic screening or intending to participate in the MSP at some time beyond the three months), and only those were categorised as women with a negative intention, who neither intended to participate in the MSP nor in opportunistic screening in the next 3 months (this category also did not include undecided women). At T3, behaviour was dichotomised as ‘participation in the MSP’ and ‘no participation in the MSP’. Women who had taken part in opportunistic screening were excluded.

Decisional conflict was measured using the 4-item SURE (Sure of myself; Understand information; Risk-benefit ratio; Encouragement) test [26]. To calculate the total score ‘uncertainty’ all items were summed (score range 0 to 4) with a high value indicating high decisional conflict. Decision regret was measured at T3 using the Decision Regret Scale [27]. Decision stage was measured with one item with the response options ‘not thought about’, ‘contemplating it’, ‘close to deciding’, and ‘choice already made’ [28].

### Statistical analysis

Data were analysed with SPSS version 23.0 (IBM, Corp., Armonk, NY) and MPlus version 7.0 (Muthen & Muthen, Los Angeles, CA). Possible baseline differences between trial arms were statistically tested with an *α* of.15. The impact of the DA on the primary outcome informed choice was analysed using cross-sectional *χ*^2^-tests for all measurement points according to the classification model of Marteau et al. [5].

All secondary outcomes with several indicators (i.e., knowledge, attitude, decisional conflict, decision regret) were modelled as latent variables which allowed to account for measurement error and to test measurement invariance. Since measurement invariance is necessary to conduct and interpret analyses on longitudinal multigroup data [29], measurement invariance levels were tested across time and group before analyses of intervention effects were performed. Partial invariance (i.e., invariance of the majority of indicators, with some parameters being freely estimated) was tested if full measurement invariance was not tenable. Partial measurement invariance is unproblematic when only few loadings or intercepts are variant [29]. Additionally, modelling outcomes as latent variables allowed us to apply full information maximum likelihood estimation enabling us to include individuals with missing values in the analysis. To test the intervention effect, autoregressive latent models [29] were applied with group (0 = control, 1 = DA) predicting T2 and T3 latent outcomes. A first order autoregressive effect of T1 on T2 and a second order autoregressive effect of T1 on T3 were specified. The autoregressive path between T2 and T3 was fixed to 0 to allow a comparison to the baseline measurement. For scale setting, all models were calculated using the Fixed-Factor-Method [29].

Two types of latent analyses were conducted: (1) Numeric secondary outcome items forming a continuous latent factor (i.e., attitude and decision regret) were analysed using confirmatory factor analysis (CFA). For these models, model fit was evaluated using the following goodness-of-fit indices: Comparative Fit Index (CFI), Tucker-Lewis Index (TLI) and Root Mean Square Error of Approximation (RMSEA). A model with acceptable fit should yield a CFI ≥ 0.90, TLI ≥ 0.90 and a RMSEA ≤ 0.08 [29]. For the CFA models, the assumption of the respective invariance level held, if the CFI difference to the model of the previous invariance level was ≤ .01 [30]. For our analyses, data had to be strong-factorially invariant (loadings and intercepts of the indicators equal over time and group) [29].

(2) Categorical secondary outcomes forming a continuous latent factor (i.e., decisional conflict and knowledge) were analysed using 2-parameter-logistic item factor analysis (IFA). For these models, the assumption of invariance held, if the loglikelihood test was not significant [31].

Single-indictor secondary outcomes were analysed with *χ*^2^-tests (intention and uptake) or a Mann-Whitney-U-test (decision stage) according to their level of measurement.

## Results

**Fig 1:**
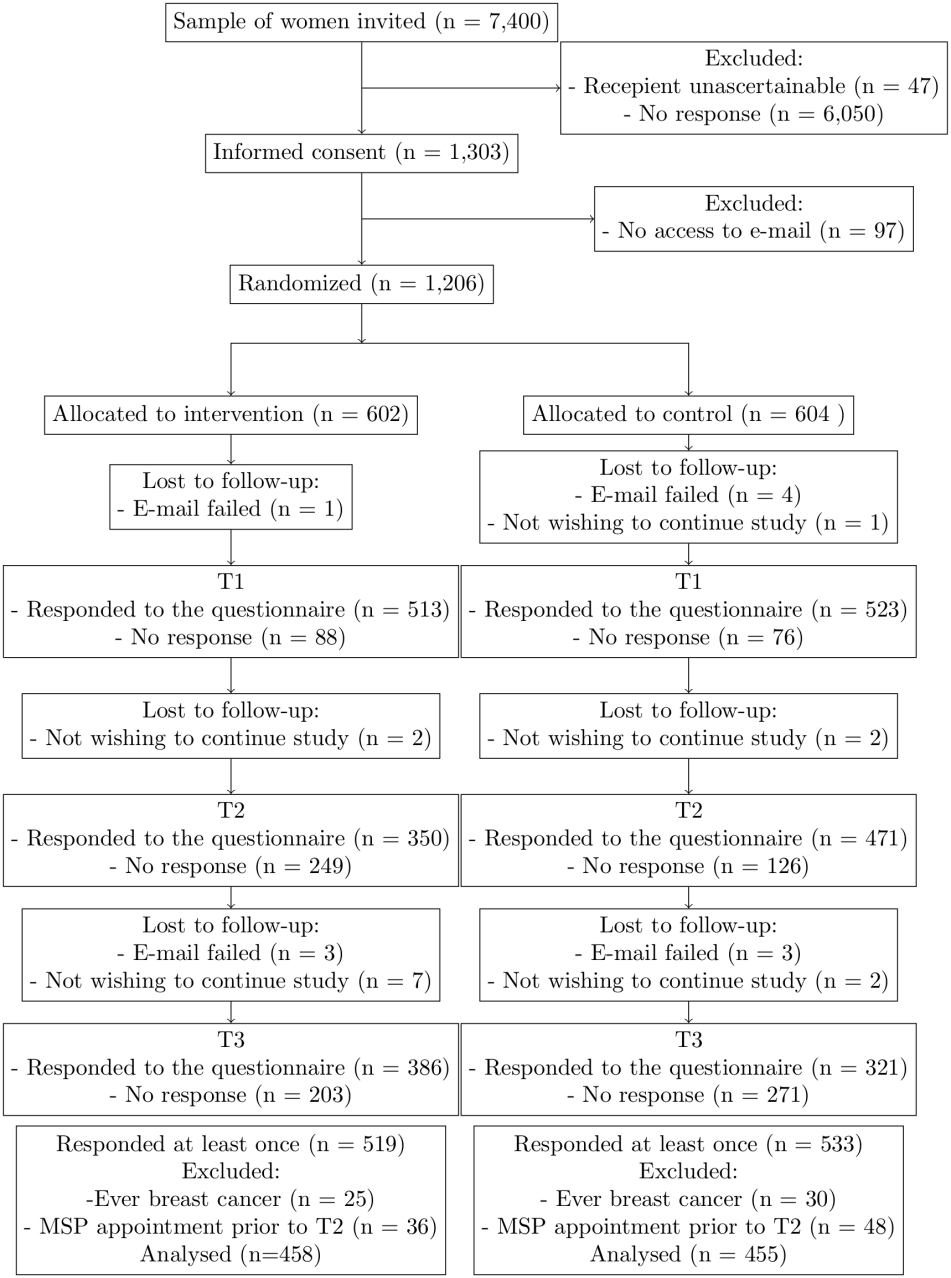
Consort flow diagram.

7,400 women were invited to take part in this study (see Fig 1). 1,206 women consented to take part in the study and provided their e-mail address, through which they were contacted at the three assessments. In our sample size calculation, we had determined a sample of 740 women [6]. Compared to our estimated response rate of 15%, the actual response rate was 16.3%. At T3 41.4% of women randomised responded. After code matching the data of all measurement points, 1,052 datasets resulted (this includes women responding at any one measurement point). Women who ever had breast cancer (n = 29), and women, who did not respond to this question (n = 26), were excluded from the analyses. Women, who self-reported at T2 that the appointment proposed in the MSP invitation already had passed, were also excluded (n = 84) because they either would already have attended the screening or decided to not attend it. Accordingly, the data of 913 women were analysed.

### Baseline characteristics

Background and outcome variables were similar between groups (Table 2). Most participating women had received at least 10 years of school education. For almost all German was their main language. More than 60% had had a mammogram in the past; of those, more than half had been conducted for screening purposes. Just under 60% had received the invitation to the MSP and the associated brochure. About 90% had a statutory health insurance covering the MSP.

**Table 2:** Baseline characteristics, n (%).

	Control	Decision aid
*General*		
Education		
9 years	43 (9.7)	50 (11.2)
10 years	189 (42.6)	194 (43.4)
11 years	74 (16.7)	57 (12.8)
≥12 years	133 (30.0)	141 (31.5)
Other	5 (1.1)	5 (1.1)
Main language		
German*	444 (99.8)	466 (100.0)
Internet information search per week		
<1h	73 (16.0)	83 (18.1)
1h to <2h	157 (34.5)	146 (31.9)
2h to <5h	150 (33.0)	139 (30.3)
5h to <10h	52 (11.4)	55 (12.0)
>10h	23 (5.1)	35 (7.6)
Internet importance		
important	264 (58.3)	263 (57.8)
neither nor	123 (27.2)	110 (24.2)
unimportant	66 (14.6)	82 (18.0)
Self-rated health		
very good	90 (20.5)	87 (19.7)
good	254 (57.7)	243 (55.0)
neither nor	82 (18.6)	98 (22.2)
bad/very bad	14 (3.2)	14 (3.2)
Mother or sister with breast cancer	70 (15.8)	63 (14.1)
*Mammography*		
Ever mammogram	284 (63.7)	289 (64.2)
If yes, reason for last mammogram:		
- screening	160 (56.7)	144 (50.2)
- diagnostic	116 (41.1)	140 (48.8)
- don’t know	6 (2.1)	3 (1.0)
Invitation to MSP received	214 (55.4)	166 (59.7)
*Health insurance*		
Health insurance:		
- Statutory health insurance**	340 (76.6)	319 (72.8)
- Statutory & complementary private health insurance	57 (12.8)	79 (18.0)
- Private health insurance***	37 (8.3)	30 (6.8)
- Other	10 (2.3)	10 (2.3)

Note.

*Including women providing more than 1 main language.

**Covers the MSP.

***Coverage of the MSP depends on the insurance.

### Primary analysis

Most women made no informed choice irrespective of time or group (see Table 3). In the control group the proportion of uninformed choices was constant at about 70%. The proportion of informed choices at T1 was similar between the groups (*χ*^2^ = 0.43, *df* = 1, *p* = .542). At T2 (*χ*^2^ = 57.20, *df* = 1, *p* < .001) and T3 (*χ*^2^ = 5.24, *df* = 1, *p* = .024) the proportion of informed choices in the DA group was significantly higher. Thus, women in the control group had at T2 (OR 0.26, 95% CI 0.18-0.37) and T3 (OR 0.66, 95% CI 0.46-0.94) lower odds, to make an informed choice than women in the DA group.

**Table 3:** Informed choice and its three dimensions, n (%).

Percentage of…	Group	T1	T2	T3
adequate knowledge	DA	129 (28.6)	189 (66.8)	141 (51.3)
	control	133 (29.8)	122 (31.4)	131 (40.2)
positive attitude	DA	407 (90.0)	235 (83.0)	229 (84.2)
	control	399 (88.7)	342 (87.5)	275 (85.1)
positive intention/completed screening	DA	295 (87.3)	190 (81.9)	168 (65.4)
	control	194 (87.5)	280 (90.0)	203 (67.4)
informed choice	DA	87 (26.0)	142 (61.5)	99 (39.8)
	control	94 (28.3)	89 (28.9)	88 (30.3)

### Secondary analysis

#### Knowledge

The proportion of women with adequate knowledge was similar between the groups at T1, and being less than one-third, this proportion must be seen as low (Table 4). Table 5 shows the model fit indices of the measurement invariance models and of the predictor model. Since item 6 (Who is more likely to die of breast Cancer? Women participating in the MSP/ Women not participating in the MSP/ Both the same) showed negative loadings (women with a higher level of knowledge were less likely to answer the item correctly), it was excluded. Since we assume in knowledge items that a correct response correspond with a higher knowledge level, negative loading or loadings close to zero are not adequate to differentiate between knowledge levels.

**Table 4:** Decisional conflict, decision regret, and decision stage, M (SD).

Outcome	Group	T1	T2	T3
Knowledge	DA	2.73 (1.41)	3.96 (1.33)	3.57 (1.16)
	Control	2.79 (1.34)	2.92 (1.40)	3.21 (1.28)
Attitude	DA	3.39 (2.91)	2.96 (3.41)	2.84 (3.51)
	Control	3.33 (2.88)	3.20 (2.94)	3.39 (3.48)
Decisional conflict	DA	1.49 (1.62)	0.52 (0.86)	0.62 (1.09)
	Control	1.55 (1.59)	0.99 (1.37)	0.86 (1.21)
n (%) of Yes-responses				
*Understand information*	DA	294 (65.9)	272 (97.8)	246 (88.8)
	Control	284 (63.7)	308 (80.4)	275 (84.1)
*Risk-benefit ratio*	DA	292 (65.3)	257 (92.1)	245 (88.8)
	Control	287 (64.3)	295 (77.0)	264 (81.0)
*Encouragement*	DA	260 (58.6)	233 (83.8)	218 (79.6)
	Control	259 (58.5)	277 (73.1)	235 (71.9)
*Sure of myself*	DA	264 (58.7)	207 (74.2)	222 (80.7)
	Control	261 (58.3)	274 (70.8)	250 (77.2)
Decision regret	DA	-	-	12.18 (15.31)
	Control	-	-	12.69 (15.92)
Decision stage	DA	3.33 (1.05)	3.67 (.71)	-
	Control	3.36 (1.019)	3.58 (.811)	-
n (%) of categories				
*Not thought about it*	DA	39 (8.5)	3 (1.1)	-
	Control	37 (8.2)	9 (2.3)	-
*Thinking about both options*	DA	82 (17.9)	30 (10.7)	-
	Control	70 (15.6)	54 (13.8)	-
*Close to making a decision*	DA	25 (5.5)	23 (8.2)	-
	Control	35 (7.8)	29 (7.4)	-
*Made a decision*	DA	311 (68.1)	224 (80.0)	-
	Control	308 (68.4)	300 (76.5)	-

**Table 5:** Fit-information of 2-parameter-logistic item factor models.

Outcome	Tested model	Loglikelihood	*df*	AIC	BIC	ΔLoglikelihood	Δ*df*	*p*	Pass?
Decisional conflict	Configural invariant	-4174.26	55	8458.53	8723.24	-	-	-	-
	Full invariant	-4212.97	25	8475.94	8596.27	77.41	30	< .001	no
	Partial invariant*	-4193.85	27	8441.70	8571.66	39.17	28	.078	yes
	DA as predictor	-3611.88	18	7259.76	7346.40	-	-	-	-
Knowledge	Configural invariant**	-6539.91	115	13309.83	13862.36	-	-	-	-
	Full invariant**	-6630.46	65	13390.91	13703.21	181.09	50	< .001	no
	Partial invariant**	-6556.33	89	13290.66	13718.27	32.83	26	.167	yes
	DA as predictor	-6073.74	47	12241.48	12467.30	-	-	-	-

Note. - Parameter not possible.

*In the DA group, the thresholds of 2 items at T2 were estimated freely.

** Convergence criterion .02.

Partially strong measurement invariance was accepted. The paths from group to the latent factor knowledge T2 (*γ* = 0.151, *p* = .010) and to the latent factor knowledge T3 (*γ* = 0.103, *p* = .034) were significant indicating that at T2 and T3, the DA group had a higher knowledge level than the control compared to T1.

#### Attitude

**Fig 2:**
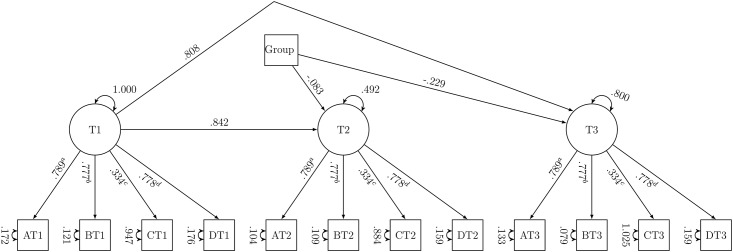
Predictor model of attitude. Unstandardised parameters; a to d: constraints over time; intercepts constrained over time and not shown; residual covariances estimated freely and not shown; A: important/unimportant, B: a good thing/a bad thing, C: pleasant/unpleasant, D: beneficial/harmful; group: 0 = control, 1 = DA.

Both groups had, at all measurement points, a very positive attitude towards participation in the MSP. Strong measurement invariance was accepted (Table 6). The path (see Fig 2) from group to the latent factor attitude T2 was not significant (*γ* = −0.083, *p* = .170). At T2, the DA group and control group had a similar attitude compared to T1. The path from group to the latent factor attitude T3 was significant (*γ* = −0.229, *p* = .004). At T3, the DA group had a more negative attitude than the control compared to T1.

**Table 6:** Fit-information of confirmatory factor models.

Outcome	Tested model	*χ*^2^	*df*	*p*	*RMSEA* (90%*CI*)	*CFI*	Δ*CFI*	*TLI*	Δ*TLI*	Pass?
Attitude	Configural invariant	105.66	78	.020	.028 (.012-.041)	.996	-	.994	-	yes
	Weak invariant	118.62	93	.038	.025 (.006-.037)	.996	.000	.995	+	yes
	Strong invariant	143.40	108	.013	.027 (.013-.038)	.995	.001	.994	.001	yes
	DA as predictor	176.31	62	< .001	.045 (.037-.053)	.984	-	.980	-	-
Decision regret	Configural invariant	65.47	10	< .001	.137 (.106-.169)	.952	-	.904	-	yes
	Weak invariant	74.27	14	< .001	.120 (.094-.148)	.948	.004	.926	+	yes
	Strong invariant	80.03	18	< .001	.108 (.084-.132)	.946	.002	.941	+	yes
	DA as predictor	61.795	9	< .001	.099 (.077-.124)	.954	-	.923	-	-

Note. - Parameter not possible. + Improvement of parameter.

#### Intention and uptake

At T1 and T2, the majority of women responded that they would participate in the MSP within the next 3 months (see Table 7). At T3, in both groups, the majority had participated in the MSP (additionally, more than 5% had conducted an opportunistic mammography). At T1, a quarter of women was undecided. For intention at T1 (*χ*^2^ = 0.01, *df* = 1, *p* = 1.000) and uptake at T3 (*χ*^2^ = 0.27, *df* = 1, *p* = .653), there were no significant differences between the groups. At T2 (*χ*^2^ = 7.56, *df* = 1, *p* = .007), there was a significant difference: The proportion of those, who did not want to participate in the screening, was higher in the DA group (18.1%) than in the control group (10.0%). Women in the control group had higher odds to have a positive intention (OR 2.00, 95% CI 1.21-3.29) than women in the DA group.

**Table 7:** Intention and uptake for DA and control, n (%).

Group	Intention	T1	T2	Uptake	T3
DA	MSP	295 (65.3)	190 (68.3)	MSP	168 (62.0)
	no MSP*	43 (9.5)	44 (15.8)	no screening	89 (32.8)
	undecided	114 (25.2)	44 (15.8)	opportunistic	14 (5.2)
Control	MSP	294 (66.1)	280 (71.8)	MSP	203 (61.9)
	no MSP*	42 (9.4)	38 (9.7)	no screening	98 (29.9)
	undecided	109 (24.5)	72 (18.5)	opportunistic	27 (8.2)

Note.

* Including women intending an opportunistic mammogram.

#### Decisional conflict

Decisional conflict was similar in the DA and control groups at T1 with average values around 1.5 on a scale of 0 to 4 (see Table 3). Partially strong measurement invariance was accepted (Table 5). The paths from group to the latent factor decisional conflict T2 (*γ* = 0.335, *p* < .001) and to the latent factor decisional conflict T3 were significant (*γ* = 0.149, *p* = .042). At T2 and T3, the DA group had lower decisional conflict than the control compared to T1.

#### Decision regret

At T3, most women experienced no regret (control: 40.8%; DA: 42.9%). Strong measurement invariance was accepted (Table 6). The path from group to T3 was not significant (*γ* = −0.060, *p* = .498). At T3, the DA group had a similar level of decision regret as the control group.

#### Decision stage

At T1 in both groups, 68% indicated they had already made a decision. This percentage increased to 76.5% in the control at T2 and to 80.0% in the DA group. For decision stage, there was neither a significant difference between groups at T1 (*U* = 101794.00, *z* = −.32, *p* = .755) nor at T2 (*U* = 52676.00, *z* = −1.23, *p* = .220).

## Discussion

This was the first study that evaluated the impact of a newly developed online DA for the German MSP in a randomised controlled trial. As hypothesised, the DA resulted in a greater proportion of informed choices, a higher knowledge level, and less decisional conflict. Contrary to our hypothesis, decision regret was not reduced by the DA. For attitude and intention/uptake, we did not formulate specific hypotheses. Our results for these outcomes were mixed: depending on the measurement point, we either found a decrease or no effect of the DA on these measures.

In the control group the proportion of uninformed choices was constant at about 70%. This confirms the results of a previous German study, according to which very few decisions about the MSP are made informed [1]. Our results at both post intervention and follow-up show that our DA led to more informed decisions than the existing information materials, that were provided with the invitation, were able to achieve alone. Previous research on levels of informed choice after exposure to a DA on mammography screening shows mixed results. Mathieu et al. (2007) evaluating a DA for women aged 70 reported a greater proportion of women making an informed choice [10] while Mathieu et al. (2010) in their cross-sectional study on women aged 40 found no difference in the proportion of women making an informed choice [13].

Regarding our secondary outcomes, the DA showed different effects. The proportion of adequate knowledge at baseline with less than one-third must be seen as low. Contrastingly, Hersch et al. reported correct responses to knowledge items of over 70% at baseline in an Australian study [18]. In a cross-sectional survey in the Netherlands, Agt et al. assessed only conceptual knowledge and reported 95% of women to have sufficient knowledge [32]. These differences may in part be explained by differences between mammography screening information materials and programmes in the different countries. Another possible explanation is that the different knowledge measures account for these wide discrepancies. Existing knowledge measures differ in content, difficulty, number of items and response formats. Our DA group had a higher knowledge level than the control group at post-intervention and follow-up. This is in line with the Cochrane review according to which DAs in general lead to higher knowledge than usual care [8]. Regarding a DA for mammography screening, Mathieu et al. (2007) found a significant increase in knowledge [10]. Similarly, Mathieu et al. (2010) also reported a significant effect of their DA on knowledge [13].

It remains unclear which component of our DA or which combination of components is responsible for the knowledge increase. Crowd figures using circles or pictograms to indicate the number of affected and unaffected people have been shown to increase accuracy of risk perception [33, 34]. DAs always interact with other factors. Petrova et al. conducted an experiment on 3 presentation formats for statistical information about breast cancer (text, fact box, visual aid) and found no main effect of information format on number of correct comprehension questions [35]. Presenting numerical information accompanied by a visual aid improved knowledge compared to alternative formats only when perceived severity of the disease was not extremely high [35]. Previous beliefs about mammography screening as well as a high level of fear of breast cancer interfere with comprehension of evidence based information and thus hinder knowledge increase and informed choices [35].

Both groups had, at all measurement points, a very positive attitude towards participation in the MSP. Hersch et al. and Agt et al. similarly reported that most women had a positive attitude [18, 32]. Post-intervention, we found no differences between groups. In contrast, at follow-up, the DA group had a more negative attitude than the control group. Our DA may have made the women feel less positive about screening through increasing their knowledge about the low personal benefit of screening, but it remains questionable why this effect did not occur at post-intervention. Similarly, Mathieu et al. reported no difference in attitude in their DA group [13].

At baseline and post-intervention, the majority of participating women responded that they would participate in the MSP within the next 3 months. At T3, in both groups, the majority had participated in the MSP. Intention to participate was lower in the DA group post-intervention. The uptake rate was unaffected by our DA regardless of a lower post-intervention intention to participate. Regarding intention and uptake, the effect of DAs is not clear. One study reported no effect on intention following a DA [36] whereas others did find a lower intention following a DA [37] or in the DA group [13]. The situation is similar for uptake with research showing no effect on uptake [10] as well as lower uptake in the DA group [24]. According to the Cochrane review, DAs with an explicit values clarification exercise were more likely to lead to a value congruent choice [8] but there is no clear effect on intention per se. Depending on the type of screening or treatment offered, more information will have a positive, no, or a negative effect. This argument similarly applies to attitude.

The proportion of women at follow-up not having participated in the MSP was significantly higher than the intention to not participate in the MSP at post-intervention. Apart from a true change of mind, other explanations have to be taken into account. We assessed intention to participate within the next 3 months. Accordingly, some women will have had the intention to take part in the next three months but either received the invitation to the MSP so late that the appointment at our third measurement point had not yet passed orsome barrier hindered an intended attendanceand the appointment was postponed. Potentially, social desirability may have been a factor influencing intention responses, since in a context where early detection is seen as something good by society, an intention to not participate may be perceived as deviant and thus not reported. Retrospective uptake responses may not be biased as strongly as screening attendance either has or has not occurred.

The DA group had lower decisional conflict than the control at both post-intervention and follow-up. This is in congruence withthe Cochrane review reporting overall, a reduction in decisional conflict following a DA [8]. Contrastingly, two randomised controlled studies on mammography screening reported no effect of a DA on decisional conflict [10, 37] whereas one study found lower decisional conflict [18]. However, this may be different for various subgroups of women resulting in opposing effects. Many women have decided before contact with a DA [38]. This then may cause more decisional conflict if personal preference and scientific evidence do not match; i.e., a DA can increase decisional conflict [38]. DAs are also seen most positive by women who merely verify their previous decision with the DA [38]. At present it is uncertain whether high decisional conflict is a good or a bad thing [24], since high decisional conflict could be an indicator for an active deliberation process [39] as well as for insufficient decision support.

Most women experienced no decision regret and this was not affected by the DA. This is in line with other research. According to the Cochrane review, only 7 studies assessed decision regret and none reported statistically significant differences [8].

At baseline, the majority of women indicated they had already made a decision which is similar to a RCT by Hersch et al. [18]. Our study groups were similar in decision stage at post-intervention and follow-up. This may be explained by our DA only being relevant for women who have not yet formed a decision. Thus, decision stage in our study would not have been expected to alter since the majority had already made the decision leaving little room for decision process progression.

### Strengths and limitations

Major strengths of this study were its design as a RCT and as one of the first the follow-up at 3 months. This allowed the evaluation of long-term effects of the DA (e.g., the persistence of increased knowledge) and also of screening uptake. Importantly, another strength of this research is that we developed and evaluated the DA in accordance with IPDAS criteria which are designed to ensure a minimum level of quality for DAs. Our DA was systematically developed and evidence based. The brochure that was in use during our study and was thus the usual care, had been in use since September 2010 and openly discussed benefits and harms of the MSP [40]. Only some but not all statistics were presented in absolute numbers [41]. Gummersbach and Abholz indicate that a graphical presentation of the numbers may have been useful and that the probable lack of effect on overall mortality should be mentioned [41]. All 3 points were all fulfilled by our DA but not by the brochure.

Most respondents received the questionnaire at about the time of receipt of the official invitation materials. This ensured a high relevance of the decision. Some women had not yet received the invitation to the MSP and the associated brochure. Thus, the timing of the decision and the availability of usual care information materials was heterogeneous. Since the information brochure is also available online, it remains speculative how whether the women not yet having received their invitation had access to it.

To avoid the potential disruptive influence of previous screening experiences or habits, only women aged 50 who are invited for the first time to the MSP were included in the study. Our study achieved acceptable response and attrition rates but it has to be noted that women participating in our study will likely have been different from those not participating: in particular, it can be assumed that they may have been more interested in mammography screening as a topic and thus may probably have informed themselves about this topic more than the general population.

Since the sample we drew from the population registries was representative except for women with Turkish migration background, our results cannot be applied to this group. We had almost no women in our sample whose main language did not include German. Women whose mother tongue was not German may have been less likely to participate in our study.

In calculating informed choice, intention to participate in the MSP within the next three months was used as one of 3 dimensions. Consequently, women whose appointment was at a later date were classified as non-intenders and an artificial mismatch (or match) between attitude and intention would be created due to the time frame of the intention question. This time limitation may explain some of the inconsistencies between attitude and intention that have been categorised as uninformed decision [1]. Nevertheless, a limitation of the period was important for the validity of the question [25]. Assessed uptake at T3, since it was self-reported, will have deviated to some degree from actual uptake.

Using latent structural equation models for our analyses had several advantages. (1) We could explicitly account for measurement error. (2) Testing measurement invariance across time and groups enabled us to establish a crucial prerequisite for testing our intervention effect. Measurement invariance entails that the same construct is measured regardless of time or group. Thus, the item specific information is unaffected by these contextual factors [29]. (3) In order to deal with missing values, we could apply full information maximum likelihood estimation allowing us to include individuals with missing values in the analysis.

The knowledge index contained only some facts about the MSP. These have been carefully selected, however, by no means cover the entire spectrum of possibly decision relevant facts. Additionally, we had to exclude one item, which did not differentiate women with a high or low knowledge level. It remains speculative, what caused this problem in this item.

### Practice implications and future research

An important aspect for future research is the influence of affect on information processing, especially since decisions about cancer screening involve counterintuitive evidence [35] like overdiagnoses. When cancer is perceived as a severe condition, understanding of screening statistics can be reduced [35]. Therefore, when the consequences of a decision are affect-rich (e.g., when women experience fear of breast cancer) heuristic processes are engaged, which neglect this probabilistic information [42]. Hence, for women in our study with strong previous beliefs, the effect of our DA may have been limited. Previous research showed that visual aids have no benefits for women regarding breast cancer as highly severe condition [35]. These women might have rather relied on established beliefs being less motivated to engage with the visual aids than women experiencing less severe affect regarding breast cancer [35]. These moderating and mediating factors will be interesting to assess in future research.

Since almost all trials on DAs fail to assess potential long-term effects on health or quality of life [24]—and our study is no exception to this—it would be interesting not only to include these measures but also to see how the different outcome groups of screening (false-positives, negatives, true positives) affect these measures. We assessed screening outcomes, but in this time span, to few events for any calculations occurred.

It can be hypothesised that the effect of the DA will be larger in women with higher health literacy. It remains questionable in how far a one size fits all approach will ever be feasible for a DA on the MSP. Not only should a DA be available in different languages and for different levels of health literacy but also for women with differing existing knowledge levels and finally with differing motivations to engage with this decision. A tailored approach therefore, may be indicated in future DA developments. Alternatively, at least regarding health literacy levels, a universal precautions approach may be beneficial although it may be difficult to keep the DA to an acceptable length. A possibility may be to further develop the DA to include more explanatory information sections that can be accessed if desired (e.g., as mouse hover boxes).

After the end of our study, the information brochure of the MSP has been updated [43] and, additionally, was further developed into a DA and a concept for a future online version [12]. The new materials have not yet been evaluated in an RCT and a comparison with our DA would be interesting.

### Conclusion

This is the first study to assess the impact of an online DA for the German MSP in a RCT. The DA developed in this project is a valuable tool to support decision making and has been implemented through making it publicly available on the website of the BARMER since December 2015. Since the DA has proven to be effective in supporting women to make an informed choice, the results are relevant to practice and the DA can be widely used to support women in decision making. The DA also significantly increased women’s knowledge about the screening and decreased decisional conflict.

## Supporting information

S1 File Original application to the ethics committee in German. (PDF)

S2 File English translation of the application to the ethics committee. (PDF)

S3 File CONSORT checklist. (PDF)

## References

[1] Berens EM, Reder M, Razum O, Kolip P, Spallek J. Informed Choice in the German Mammography Screening Program by Education and Migrant Status: Survey among First-Time Invitees. PLoS One. 2015;10(11):e0142316 doi: 10.1371/journal.pone.0142316 26529513 10.1371/journal.pone.0142316 PMC4631499

[2] Stefanek ME. Uninformed compliance or informed choice? A needed shift in our approach to cancer screening. Journal of the National Cancer Institute. 2011;103(24):1821–1826. doi: 10.1093/jnci/djr474 22106094 10.1093/jnci/djr474

[3] Gøtzsche PC, Jørgensen KJ. Screening for breast cancer with mammography. Cochrane Database Syst Rev. 2013;6:CD001877. 10.1002/14651858.CD001877.pub5 PMC6464778 23737396

[4] Gigerenzer G, Mata J, Frank R. Public Knowledge of Benefits of Breast and Prostate Cancer Screening in Europe. Journal of the National Cancer Institute. 2009;101(17):1216–1220. doi: 10.1093/jnci/djp237 19671770 10.1093/jnci/djp237 PMC2736294

[5] Marteau TM, Dormandy E, Michie S. A measure of informed choice. Health Expectations. 2001;4(2):99–108. doi: 10.1046/j.1369-6513.2001.00140.x 11359540 10.1046/j.1369-6513.2001.00140.x PMC5060053

[6] Reder M, Kolip P. Does a decision aid improve informed choice in mammography screening? Study protocol for a randomized controlled trial. BMC Womens Health. 2015;15:53 doi: 10.1186/s12905-015-0210-5 26198675 10.1186/s12905-015-0210-5 PMC4510898

[7] Jørgensen KJ. Mammography screening. Benefits, harms, and informed choice. Dan Med J. 2013;60(4):B4614 23651722

[8] Stacey D, Légaré F, Col NF, Bennett CL, Barry MJ, Eden KB, et al Decision aids for people facing health treatment or screening decisions. Cochrane Database Syst Rev. 2014;1:CD001431. 10.1002/14651858.CD001431.pub4 24470076

[9] Volk RJ, Llewellyn-Thomas H, Stacey D, Elwyn G. Ten years of the International Patient Decision Aid Standards Collaboration: evolution of the core dimensions for assessing the quality of patient decision aids. BMC Med Inform Decis Mak. 2013;13 Suppl 2:S1 doi: 10.1186/1472-6947-13-S2-S1 10.1186/1472-6947-13-S2-S1 PMC4044280 24624947

[10] Mathieu E, Barratt A, Davey HM, McGeechan K, Howard K, Houssami N. Informed Choice in Mammography Screening: A Randomized Trial of a Decision Aid for 70-Year-Old Women. Archives of Internal Medicine. 2007;167(19):2039–2046. doi: 10.1001/archinte.167.19.2039 17954796 10.1001/archinte.167.19.2039

[11] Lenz M, Buhse S, Kasper J, Kupfer R, Richter T, Mühlhauser I. Decision aids for patients. Dtsch Arztebl Int. 2012;109(22-23):401–8. doi: 10.3238/arztebl.2012.0401 22778792 10.3238/arztebl.2012.0401 PMC3389744

[12] Institut für Qualität und Wirtschaftlichkeit im Gesundheitswesen. Einladungsschreiben und Entscheidungshilfe zum Mammographie-Screening. Institut für Qualität und Wirtschaftlichkeit im Gesundheitswesen; 2016. Available from: https://www.iqwig.de/download/P14-03_Abschlussbericht_Einladungsschreiben-und-Entscheidungshilfe-zum-Mammographie-Screening.pdf [cited 24 February 2017].

[13] Mathieu E, Barratt AL, McGeechan K, Davey HM, Howard K, Houssami N. Helping women make choices about mammography screening: An online randomized trial of a decision aid for 40-year-old women. Patient Education and Counseling. 2010;81(1):63–72. doi: 10.1016/j.pec.2010.01.001 20149953 10.1016/j.pec.2010.01.001

[14] Ottawa Hospital Research Institute. A to Z Inventory of Decision Aids; 2014. Available from: https://decisionaid.ohri.ca/AZinvent.php [cited 04. January 2017].

[15] Weill Cornell Medical College. Breast Screening Decisions—A mammogram decision aid for women ages 40-49; 2014. Available from: http://breastscreeningdecisions.com [cited 06. January 2017].

[16] Healthwise Incorporated. Breast Cancer Screening and Dense Breasts: What Are My Options?; 2016. Available from: https://www.healthwise.net/cochranedecisionaid/Content/StdDocument.aspx?DOCHWID=abp1927 [cited 06. January 2017].

[17] Healthwise Incorporated. Breast Cancer Screening: When Should I Start Having Mammograms?; 2016. Available from: https://www.healthwise.net/cochranedecisionaid/Content/StdDocument.aspx?DOCHWID=abh0460 [cited 06. January 2017].

[18] Hersch J, Barratt A, Jansen J, Irwig L, McGeechan K, Jacklyn G, et al Use of a decision aid including information on overdetection to support informed choice about breast cancer screening: a randomised controlled trial. Lancet. 2015;385(9978):1642–52. doi: 10.1016/S0140-6736(15)60123-4 25701273 10.1016/S0140-6736(15)60123-4

[19] Eden KB, Scariati P, Klein K, Watson L, Remiker M, Hribar M, et al Mammography Decision Aid Reduces Decisional Conflict for Women in Their Forties Considering Screening. J Womens Health (Larchmt). 2015;24(12):1013–20. doi: 10.1089/jwh.2015.5256 26360918 10.1089/jwh.2015.5256 PMC4683542

[20] Gemeinsamer Bundesausschuss, Stabsbereich Öffentlichkeitsarbeit und Kommunikation. Informationen zum Mammographie-Screening: Programm zur Früherkennung von Brustkrebs für Frauen zwischen 50 und 69 Jahren; 2010. Available from: http://www.mammo-programm.de/download/merkblatt_deutsch_web.pdf [cited 14 January 2014].

[21] Berens EM, Reder M, Kolip P, Spallek J. A cross-sectional study on informed choice in the mammography screening programme in Germany (InEMa): A study protocol. BMJ Open. 2014;4(9):e006145 doi: 10.1136/bmjopen-2014-006145 25231495 10.1136/bmjopen-2014-006145 PMC4166244

[22] Razum O, Zeeb H, Beck K, Becher H, Ziegler H, Stegmaier C. Combining a name algorithm with a capture-recapture method to retrieve cases of Turkish descent from a German population-based cancer registry. Eur J Cancer. 2000;36(18):2380–2384. doi: 10.1016/S0959-8049(00)00333-6 11094313 10.1016/s0959-8049(00)00333-6

[23] Elwyn G, O’Connor A, Stacey D, Volk R, Edwards A, Coulter A, et al Developing a quality criteria framework for patient decision aids: online international Delphi consensus process. BMJ. 2006;333(7565):417 doi: 10.1136/bmj.38926.629329.AE 16908462 10.1136/bmj.38926.629329.AE PMC1553508

[24] Smith SK, Trevena L, Simpson JM, Barratt A, Nutbeam D, McCaffery KJ. A decision aid to support informed choices about bowel cancer screening among adults with low education: randomised controlled trial. BMJ. 2010;341:c5370 doi: 10.1136/bmj.c5370 20978060 10.1136/bmj.c5370 PMC2965151

[25] Fishbein M, Ajzen I. Predicting and changing behavior: The reasoned action approach. New York: Psychology Press; 2010.

[26] Légaré F, Kearing S, Clay K, Gagnon S, D’Amours D, Rousseau M, et al Are you SURE?: Assessing patient decisional conflict with a 4-item screening test. Can Fam Physician. 2010;56(8):e308–14. 20705870 PMC2920798

[27] Brehaut JC, O’Connor AM, Wood TJ, Hack TF, Siminoff L, Gordon E, et al Validation of a decision regret scale. Med Decis Making. 2003;23(4):281–292. doi: 10.1177/0272989X03256005 12926578 10.1177/0272989X03256005

[28] O’Connor A. User Manual—Stage of Decision Making; 2000 [updated 2003]. Available from: http://decisionaid.ohri.ca/docs/develop/User_Manuals/UM_Stage_Decision_Making.pdf [cited 20 Ocober 2016].

[29] Little PTD. Longitudinal structural equation modeling. New York, NY: Guilford Press; 2013.

[30] Cheung GW, Rensvold RB. Evaluating Goodness-of-Fit Indexes for Testing Measurement Invariance. Structural Equation Modeling: A Multidisciplinary Journal. 2002;9(2):233–255. doi: 10.1207/S15328007SEM0902_5

[31] Hoffman L. Latent Trait Measurement Models for Binary Responses: IRT and IFA; 2014. Available from: http://www.lesahoffman.com/PSYC948/948_Lecture6_Binary_Responses.pdf [cited 26 March 2017].

[32] van Agt H, Fracheboud J, van der Steen A, Koning Hd. Do women make an informed choice about participating in breast cancer screening? A survey among women invited for a first mammography screening examination. Patient Education and Counseling. 2012;89(2):353–359. doi: 10.1016/j.pec.2012.08.003 22963769 10.1016/j.pec.2012.08.003

[33] Galesic M, Garcia-Retamero R, Gigerenzer G. Using icon arrays to communicate medical risks: overcoming low numeracy. Health Psychology. 2009;28(2):210 doi: 10.1037/a0014474 19290713 10.1037/a0014474

[34] Garcia-Retamero R, Galesic M. Who profits from visual aids: overcoming challenges in people’s understanding of risks [corrected]. Soc Sci Med. 2010;70(7):1019–25. doi: 10.1016/j.socscimed.2009.11.031 20116159 10.1016/j.socscimed.2009.11.031

[35] Petrova D, Garcia-Retamero R, Cokely ET. Understanding the Harms and Benefits of Cancer Screening: A Model of Factors That Shape Informed Decision Making. Med Decis Making. 2015;35(7):847–58. doi: 10.1177/0272989X15587676 26044208 10.1177/0272989X15587676

[36] Scariati P, Nelson L, Watson L, Bedrick S, Eden KB. Impact of a decision aid on reducing uncertainty: pilot study of women in their 40s and screening mammography. BMC Med Inform Decis Mak. 2015;15:89 doi: 10.1186/s12911-015-0210-2 26554555 10.1186/s12911-015-0210-2 PMC4640415

[37] Schonberg MA, Hamel MB, Davis RB, Griggs MC, Wee CC, Fagerlin A, et al Development and evaluation of a decision aid on mammography screening for women 75 years and older. JAMA Intern Med. 2014;174(3):417–24. doi: 10.1001/jamainternmed.2013.13639 24378846 10.1001/jamainternmed.2013.13639 PMC4017368

[38] Elisabeth Müller V, Schmacke N, Kolip P, Berger B. [Desirable, unfamiliar and in need of communication—the evidence-based decision aid of the Institute for Quality and Efficiency in Health Care (IQWiG)]. Z Evid Fortbild Qual Gesundhwes. 2012;106(4):290–4. 22749077 10.1016/j.zefq.2012.03.001

[39] Nelson WL, Han PKJ, Fagerlin A, Stefanek M, Ubel PA. Rethinking the objectives of decision aids: a call for conceptual clarity. Med Decis Making. 2007;27(5):609–18. doi: 10.1177/0272989X07306780 17873251 10.1177/0272989X07306780

[40] Gummersbach E, in der Schmitten J, Abholz HH, Wegscheider K, Pentzek M. Effects of different information brochures on women’s decision-making regarding mammography screening: study protocol for a randomized controlled questionnaire study. Trials. 2013;14:319 doi: 10.1186/1745-6215-14-319 24083811 10.1186/1745-6215-14-319 PMC3851440

[41] Gummersbach E, Abholz HH. Neues Merkblatt für Mammografie-Screening in Deutschland: Hilfe zur Meinungsbildung der Eingeladenen. Zeitschrift für Allgemeinmedizin. 2011; p. 21–25.

[42] Pachur T, Hertwig R, Wolkewitz R. The affect gap in risky choice: Affect-rich outcomes attenuate attention to probability information. Decision. 2014;1(1):64 doi: 10.1037/dec0000006

[43] Federal Joint Committee (Gemeinsamer Bundesausschuss, G-BA). Information about Mammography Screening: Program for early breast cancer detection for women aged between 50 and 69; 2015. Available from: http://www.mammo-programm.de/download/downloads/broschueren/GBA_MAMMO_ENGLI_20160310_web.pdf [cited 27 January 2017].

