# Supplementary material for: Does a decision aid improve informed choice in mammography screening? Results from a randomised controlled trial
Source: PLoS One. 2017 Dec 13;12(12):e0189148. doi: 10.1371/journal.pone.0189148 (PMC5728514; doi:10.1371/journal.pone.0189148)
Supplement: S1 File — (PDF) [file pone.0189148.s001.pdf]

Antrag auf Begutachtung eines biomedizinischen Forschungsvorhabens am Menschen durch die Ethikkommission der Medizinischen Fakultät der Westfälischen Wilhelms-Universität

1 Formale Angaben

1.1 Datum der Antragstellung

01.05.2013

1.2 Titel des Vorhabens

„Entwicklung und Testung einer Entscheidungshilfe zur Teilnahme am Mammographie-Screening-Programm (EnTeMa)“

1.3 Angaben zur Person des verantwortlichen Projektleiters

1.3.1 Name, Vorname, akademische Grade, Dienststellung

Kolip, Petra, Prof. Dr.

1.3.2 Adresse und Telefonnummer, ggf. Fax (für Rückfragen bezüglich des Antrags)

Universität Bielefeld
Fakultät für Gesundheitswissenschaften
AG für Prävention und Gesundheitsförderung
Universitätsstr. 25
33615 Bielefeld

Tel.: 0521/ 106 67273
Fax: 0521/ 106 6433
Email: petra.kolip@uni-bielefeld.de

1.3.3 Ort und Zeitpunkt der Erteilung der Approbation

Für diesen Antrag nicht relevant.

1.3.4 Ort und Zeitpunkt der Erteilung der Berufsausübungsgenehmigung für die Bundesrepublik Deutschland (nur bei Ausländern)

Für diesen Antrag nicht relevant.

1.3.5 Bei Arzneimittelprüfungen: Kann der Projektleiter eine mindestens zweijährige Erfahrung in der klinischen Prüfung von Arzneimitteln nachweisen?

Für diesen Antrag nicht relevant.

1.4 Wird die Studie in Zusammenarbeit mit weiteren Untersuchern durchgeführt? (ggf. Angaben wie zu 1.3.1.-1.3.4.)

Die Studie wird in Kooperation mit dem Projekt „Informierte Entscheidung deutscher und türkischer Frauen bei der Teilnahme am Mammographie-Screening-Programm (InEMa)" durchgeführt. Für dieses Vorhaben liegt bereits ein positives Votum vor. Projektleiter sind Jun.Prof. Dr. Jacob Spallek und Prof. Dr. Petra Kolip. Eine Absichtserklärung zur Kooperation findet sich im Anhang.

1.4.1 Name, Vorname, akademische Grade, Dienststellung

Für diesen Antrag nicht relevant.

1.4.2 Adresse und Telefonnummer, ggf. Fax (für Rückfragen bezüglich des Antrags)

Für diesen Antrag nicht relevant.

1.4.3 Ort und Zeitpunkt der Erteilung der Approbation

Für diesen Antrag nicht relevant.

1.4.4 Ort und Zeitpunkt der Erteilung der Berufsausübungsgenehmigung für die Bundesrepublik Deutschland (nur bei Ausländern)

Für diesen Antrag nicht relevant.

1.3.5 Bei Arzneimittelprüfungen: Kann der Projektleiter eine mindestens zweijährige Erfahrung in der klinischen Prüfung von Arzneimitteln nachweisen?

Für diesen Antrag nicht relevant.

1.5 Angaben zur Forschungsstelle

1.5.1 Bezeichnung des Instituts, der Klinik, der Abteilung bzw. der Praxis

AG für Prävention und Gesundheitsförderung, Fakultät für Gesundheitswissenschaften, Universität Bielefeld

1.5.2 Direktor/Leiter

Prof. Dr. Phil. Petra Kolip

1.5.3 Wird das Projekt in Zusammenarbeit mit weiteren Forschungsstellen (insbesondere bei Multi-Center-Studien) durchgeführt? (Wenn ja, bezeichnen Sie bitte die Forschungsstelle, deren Direktor/ Leiter, den dort verantwortlichen Projektleiter sowie den Koordinator der gesamten Studie.)

Für diesen Antrag nicht relevant.

1.6 Angaben zur Finanzierung

1.6.1 Bitte benennen Sie den Initiator der Studie (Projektleiter/ externe Auftraggeber, z.B. Industrie, andere)

Initiiert wurde die Studie von Prof. Dr. Petra Kolip in Kooperation mit JunProf. Dr. Jacob Spallek. Angaben zur Finanzierung unter 1.6.2

1.6.2 Durch wen wird das Projekt finanziert?

Das Projekt wird durch die Universität Bielefeld finanziert.

2 Inhaltliche Angaben

2.1 Angaben zum Forschungsvorhaben

Das Projekt wird in Kooperation mit dem Projekt InEMa (Jun.Prof. Dr. Jacob Spallek, Prof. Dr. Petra Kolip) durchgeführt (Absichtserklärung siehe Anhang). Bestimmte Passagen, die auf beide Studien zutreffen, wurden aus dem InEMa-Ethikantrag übernommen.

2.1.1 Ziel der Studie (Warum wird die Studie durchgeführt? Was ist bekannt? Vorstudien? Was soll erforscht werden?)

Entscheidungshilfen sind Interventionen, um informierte Entscheidungen zu realisieren. Sie werden bereits in zahlreichen gesundheitsrelevanten Entscheidungskontexten eingesetzt. Diese Studie soll klären, ob eine Entscheidung informierter ist, wenn eine Entscheidungshilfe über Mammographie-Screening genutzt wurde und ob demographische Faktoren einen Einfluss haben.

Folgende Fragestellungen sollen hierbei untersucht werden:

1. Erhöht eine Entscheidungshilfe den Anteil informierter Entscheidungen?
2. Wie wirkt eine Entscheidungshilfe auf die Komponenten der informierten Entscheidung (Wissen, Werte/Einstellung, Intention, Implementierung der Intention)?
3. Erhöht eine Entscheidungshilfe die Zufriedenheit mit der Entscheidung?
4. Unterscheidet sich der Effekt der Entscheidungshilfe in Abhängigkeit von bestimmten demographischen Faktoren, verschiedenen Outcome Gruppen des Screenings und der mit der Entscheidungshilfe verbrachten Zeit?

In dieser Studie wird der sozioökonomische Status analog zur Studie zur Gesundheit Erwachsener in Deutschland (DEGS) (Robert Koch-Institut) erfasst. Dies hat den Vorteil, dass Referenzdaten zum sozioökonomischen Status vorliegen.

2.1.2 Ist bekannt, ob an anderer Stelle Studien mit demselben oder ähnlichem Ziel durchgeführt wurden oder werden?

Die Studie InEMa erhebt informierte Entscheidung mit Hilfe des gleichen Messinstruments, ohne dass in InEMa eine Entscheidungshilfe eingesetzt wird.

2.1.3 Ablauf der Studie? (Bitte beschreiben Sie so präzise wie möglich den Gegenstand, den Studienablauf, die Methodik, die Dosierungs- und Anwendungsweise, die Art der Applikation etc., ggf. mit Hilfe einer schematischen Darstellung)

Das Instrument zur Messung informierter Entscheidung im MSP wurde bereits im Rahmen der Studie InEMa entwickelt. Die Entscheidungshilfe wird auf Grundlage evidenzbasierter Daten und nach dem Vorbild bereits bestehender Entscheidungshilfen für Frauen unter 50 und über 70 aus anderen Ländern, sowie Entscheidungshilfen aus anderen Screening-Kontexten

entworfen. Das Messinstrument wird zu zwei Erhebungszeitpunkten eingesetzt: kurz nach Erhalt der Einladung zum Screening (T1) sowie etwa einen Monat nach dem MSP-Termin (T2). Die Entscheidungshilfe wird zum ersten Erhebungszeitpunkt zur Verfügung gestellt.

Die Studie umfasst folgende Arme:

1. Kontrollgruppe „Online-Befragung“: Webbasierter Fragebogen
2. Experimentalgruppe „Entscheidungshilfe“: Webbasierter Fragebogen, webbasierte Entscheidungshilfe

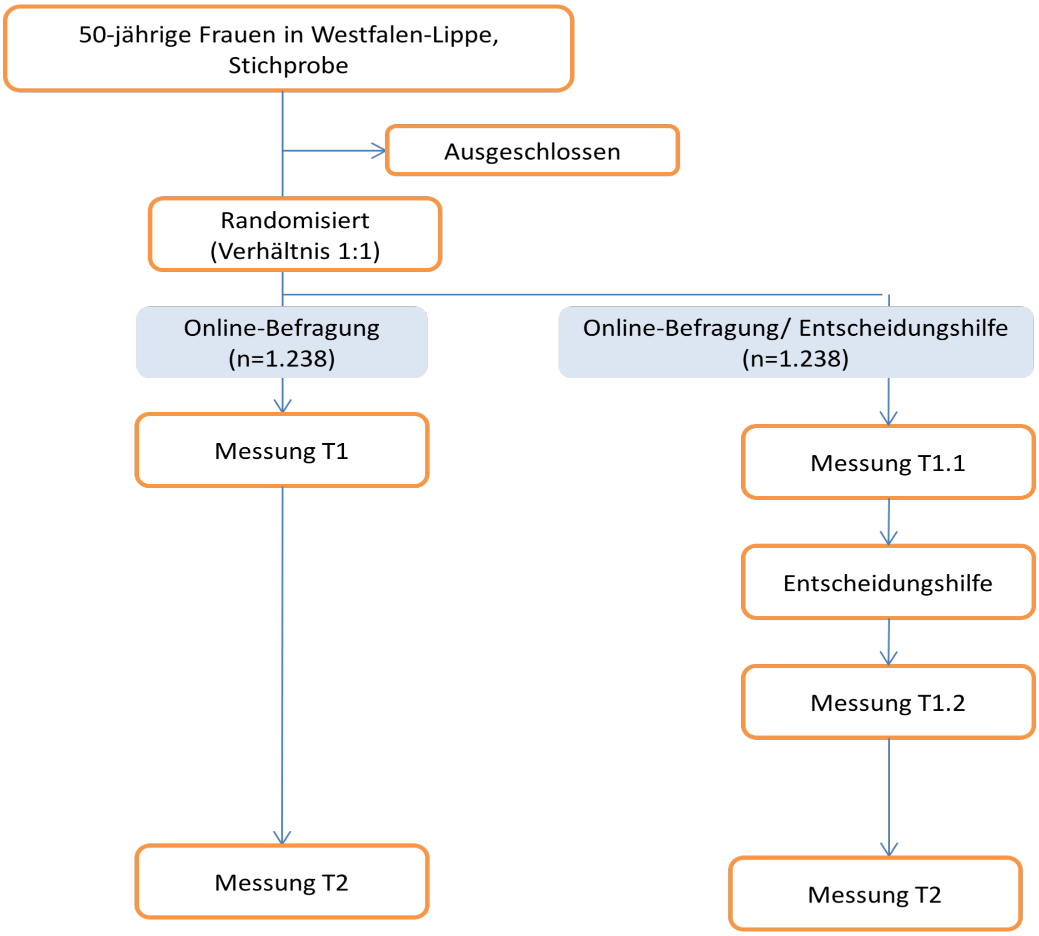

Abbildung 1: Übersicht über die Studienarme „Online-Befragung“ und „Entscheidungshilfe“.

Sowohl Wissen als auch Einstellung werden zu beiden Messzeitpunkten erfasst, um den Effekt der Entscheidungshilfe bewerten zu können. Die Intention wird zu T1 erfasst, die selbstberichtete retrospektive Teilnahme zu T2.

Eine Entscheidungshilfe für erstmalig zum Mammographie-Screening-Programm eingeladene Frauen in Deutschland wird entwickelt. Diese beinhaltet Informationen über das Mammographie-Screening, Beispiel-Arbeitsblätter und ein persönliches Arbeitsblatt zu persönlichen Werten und zur Entscheidung zur (Nicht-)Teilnahme. Die Frauen im Arm „Entscheidungshilfe“ verfügen über ein Informations- und Entscheidungs-Tool zusätzlich zu der Informationsbroschüre „Früherkennung von Brustkrebs. Was Sie darüber wissen sollten“ (Kooperationsgemeinschaft Mammographie, Deutsches Krebsforschungszentrum - Krebsinformationsdienst, 2009). In der Entscheidungshilfe werden keine anderen

Informationen dargeboten als ohnehin in der Broschüre enthalten sind. Lediglich die Präsentation und die Interaktionsmöglichkeiten mit dem Material unterscheiden sich. Die Wahrscheinlichkeit der verschiedenen Outcomes wird als Anzahl an Ereignissen pro 200 Frauen über 20 Jahre, ergänzt durch ein Crowd-Figure-Pictogram, dargestellt. Die Entscheidungshilfe wird zunächst in einem Pre-Test mit 100 Frauen überprüft.

Anspruchsberechtigte Frauen im Alter von 50 Jahren in einer Screeningregion werden Online befragt. Die Stichprobe der Studienteilnehmerinnen wird repräsentativ aus den Daten der Einwohnermeldeämter im Verwaltungsgebiet der Kassenärztlichen Vereinigung Westfalen-Lippe gezogen.

In der geplanten Studienregion, dem Bezirk der Kassenärztlichen Vereinigung Westfalen-Lippe, gibt es 13 Screening-Einheiten. Damit der Bevölkerungsbezug im Mammographie-Screening-Programm gewährleistet werden kann, werden termin- und ortsbezogene Einladungen auf Basis der Einwohnermeldedaten über so genannte Zentrale Stellen versandt. Die zentralen Stellen generieren aus den Adressdaten der anspruchsberechtigten Frauen Erinnerungsschreiben mit Angabe von Untersuchungstermin und -ort. Mit der Einrichtung der Zentralen Stellen werden die persönlichen Daten der anspruchsberechtigten Frauen rechtlich, räumlich und personell von den Leistungserbringern getrennt. Zur Gewährleistung des Datenschutzes werden im Rahmen der Studie eigenständige Stichproben 50- jähriger Frauen in den entsprechenden Einwohnermeldeämtern gezogen. Eine Nutzung der Daten der Zentralen Stellen findet nicht statt.

c) Wahl der Stichprobe und Stichprobengröße

Die zufällige Stichprobe wird über die Einwohnermeldeämter eigens für die Studie gezogen. In die Ziehung der Stichprobe werden alle Frauen im Alter von 50 Jahren einbezogen, da diese erstmals Anspruch auf ein Brustkrebsscreening im Rahmen des MSP haben. Frauen, die bereits eine Brustkrebsdiagnose hatten, werden aus der Analyse ausgeschlossen, da diese als bereits erkrankte Frauen nicht Zielgruppe des MSP sind.

In die beiden Arme der Studie sollen jeweils 1.238 Frauen eingeschlossen werden. Eine Änderung im Anteil informierter Entscheidungen sowie ein Wissenszuwachs werden mit einer Effektgröße von .1 erwartet. Die erforderliche Gesamtstichprobengröße gesetzt einer Typ I Fehlerrate von 5% und einer Typ II Fehlerrate von 20% beträgt N=2.474. Da mit einem Gesamtresponse von 10% gerechnet wird, werden 24.740 Frauen zur Studienteilnahme eingeladen. Die Rekrutierung erfolgt dabei in mehreren Wellen, je nachdem wie schnell die erforderliche Teilnehmeranzahl erreicht wird.

d) Rekrutierung / Durchführbarkeit

Den Frauen wird eine ausführliche Information über die Studie zugesendet. Die Frauen erhalten mit der Studieninformation eine Einverständniserklärung mit frankiertem Rückumschlag sowie den Link zum ersten Fragebogen und gegebenenfalls der Entscheidungshilfe.

e) Intervention

Im Arm „Entscheidungshilfe“ wird eine Entscheidungshilfe angeboten, die die gleichen Informationen beinhaltet wie die Broschüre „Früherkennung von Brustkrebs. Was Sie darüber wissen sollten“ (Kooperationsgemeinschaft Mammographie & Deutsches Krebsforschungszentrum - Krebsinformationsdienst, 2009).

f) Sekundärdatenanalyse

Sekundärdatenanalysen sind zum Vergleich mit dem Kooperationsprojekt InEMa geplant. Hier interessiert besonders, ob sich Unterschiede in der Response-Rate auf Grund des Befragungsmodus zeigen (Paper-and-pencil vs. Online).

g) Kontrolle potentieller Störgrößen
Für die Befragung wird eine Zufallsstichprobe der eingeladenen Frauen auf der Basis der Meldedaten gezogen. Bei dem gewählten Design handelt es sich um einen Randomized Controlled Trial.

Ein potentieller Störfaktor für die Studienergebnisse sind vorherige Screeningerfahrungen der Frauen. Für die Studie werden darum lediglich Frauen im Alter von 50 Jahren befragt, die zum ersten Mal für das Mammographie-Screening eingeladen werden. Zudem wird nach Vorerfahrungen im Bereich Brustkrebsfrüherkennung sowie vorliegenden Brustkrebserkrankung gefragt.

Ein mögliches Problem bei der Online Befragung einer Bevölkerungsstichprobe ist eine niedrige und selektive Teilnahmerate. In den statistischen Analysen kann für diverse mögliche erhobene Störgrößen (confounder) durch die Anwendung stratifizierter und multivariater Verfahren adjustiert werden.

h) Statistische Analysen/ Auswertung
Es werden deskriptive Auswertungen zur Beschreibung der Studienpopulation und der einzelnen sozialen Gruppen durchgeführt. Um den Arm „Entscheidungshilfe“ mit dem Arm „Online-Befragung“ zu vergleichen, kommen statistische Verfahren zum Gruppenvergleich zum Einsatz (z.B. ANOVA, chi²-Test). Multivariate Zusammenhänge werden mit Hilfe linearer und logistischer Regression überprüft.

2.1.3.1 In welcher Form wird die Studie durchgeführt? Handelt es sich um
- **eine pharmakologische, klinisch-pharmakologische, klinische oder sonstige biomedizinische Studie?**
- **eine offene oder Einfach- oder Doppelblind-Studie?**
- **randomisierte Studie?**
- **diagnostische, therapeutische, Verträglichkeits- oder ausschließlich wissenschaftliche Studie?**

Bei dieser Studie handelt es sich um einen Randomized Controlled Trial. Die Stichprobe wird randomisiert entweder dem Arm „Online-Befragung“ oder dem Arm „Entscheidungshilfe“ zugewiesen.

2.1.3.2 Von wem wird der Versuchsteilnehmer vor, während und nach der Studie ärztlich betreut? (Abstimmung mit dem Hausarzt, Kontrolle anderer Medikationen?)

Eine ärztliche Betreuung ist nicht erforderlich.

2.1.3.3 Dauer der Studie

Die Studie ist auf 3 Jahre angelegt. Die Studienvorbereitung (insbesondere Literaturrecherche und Fragebogenentwicklung) begann im Rahmen des Projekts InEMa bereits im Oktober 2012. Die Studienlaufzeit endet im September 2015.

2.1.3.4 Werden Zwischenergebnisse ausgewertet, um einen Trend zu erkennen? Nach welchen Kriterien werden welche Konsequenzen für die Teilnehmer gezogen?

Die Entscheidungshilfe wird nach der Entwicklung veröffentlicht. Eine Änderung des Forschungsvorhabens während der Durchführung ist nicht vorgesehen, da sie nach einem sorgfältigen Pre-Test und angesichts der Stichprobengröße nicht sinnvoll ist. Es werden keine negativen Auswirkungen auf die Studienteilnehmerinnen erwartet, da die Entscheidungshilfe keine anderen Informationen enthält als die oben beschriebene Broschüre, die die Frauen ohnehin zusammen mit der Einladung zum MSP erhalten.

2.1.3.5 Welche Art der Dokumentation ist vorgesehen? (Dokumentationsbögen ggf. in Kopie beifügen)

Für diesen Antrag nicht relevant.

2.1.3.6 Ist die Mitarbeit eines Statistikers vorgesehen und welche statistischen Methoden sollen benutzt werden?

Die oben beschriebenen statistischen Verfahren werden eingesetzt. Die Mitarbeit eines Statistikers ist nicht vorgesehen, da die Studienmitarbeiterinnen selbst über Erfahrungen in der statistischen Auswertung von RCTs verfügen.

2.1.3.7 Werden die ärztliche Schweigepflicht und die Datenschutzbestimmungen beachtet?

Der Umgang mit den Daten entspricht den Datenschutzbestimmungen des Landes Nordrhein-Westfalen. Eine positive Stellungnahme des Datenschutzbeauftragten der Universität Bielefeld liegt vor (siehe Anlagen).

2.1.4 Soll den Versuchsteilnehmern ein Entgelt (Aufwandsentschädigung o.ä.) gezahlt werden? In welcher Höhe?

Die Studienteilnehmerinnen erhalten keine Aufwandsentschädigung und keine Incentives.

2.1.5 Soll den beteiligten Prüfärzten ein Entgelt gezahlt werden? In welcher Höhe?

Für diesen Antrag nicht relevant.

2.2 Angaben zu den Versuchsteilnehmern

2.2.1 Anzahl (bei vergleichenden Studien bitte Aufteilung auf Gruppen angeben)

Ein Pretest der Entscheidungshilfe mit 100 Frauen wird durchgeführt.

In der tatsächlichen Studie sollen insgesamt 2.476 Frauen einbezogen werden, je 1.238 in der Kontroll- und Experimentalgruppe.

2.2.2 Alter und Geschlecht (bitte geben Sie das Alter der Versuchsteilnehmer sowie die als Ausschlußkriterien vorgesehenen Ober- und Untergrenzen an)

Es werden ausschließlich Frauen im Alter von 50 Jahren befragt, da diese erstmalig eine Einladung zum Mammographie-Screening erhalten.

2.2.3 Status: Handelt es sich bei den Versuchsteilnehmern um
a) gesunde Personen?
b) schwangere oder stillende Frauen?
c) einschlägig Erkrankte? (bitte geben Sie die Krankheit und das Stadium an)
d) stationäre oder ambulante Patienten?
e) Personen, die an anderen Krankheiten leiden? (insbesondere psychische Krankheiten, die Zweifel an der Geschäfts- oder Einsichtsfähigkeit begründen?)

Die Studienteilnehmerinnen werden nicht nach ihrem Gesundheitszustand zur Teilnahme an der Studie ausgewählt.

2.2.4 Welche sonstigen Einschlußkriterien (z.B. erlaubte Begleitmedikation) sind vorgesehen?

Keine.

2.2.5 Welche sonstigen Ausschlußkriterien (z.B. fortgeschrittene Nieren- oder Leberinsuffizienz, verbotene Begleitmedikation etc.) sind vorgesehen?

Keine.

2.2.6 Sollen Personen teilnehmen, die auf gerichtliche oder behördliche Anordnung in einer Anstalt verwahrt werden?

Nein.

2.2.7 Sollen Personen teilnehmen, die sich schon für andere Forschungsvorhaben zur Verfügung gestellt haben (ggf. wann, wie oft)?

Nein.

2.3 Angaben zur Nutzen-Risiko-Relation

2.3.1 Welcher Nutzen ist von den Ergebnissen der Studie zu erwarten
a) für die Versuchsteilnehmer?

Für die Teilnehmerinnen der Experimentalgruppe ist ein höherer Anteil an informierten Entscheidungen zu erwarten.

b) für die Heilkunde?

Kein direkter Nutzen.

c) für die Wissenschaft (z.B. Ergebnisse, die nicht unmittelbar therapeutischen Zwecken dienen)?

Übergeordnetes Ziel dieser Studie ist es, einen Beitrag dazu zu leisten, den Anteil an informierten Entscheidungen für oder gegen die Teilnahme am MSP zu erhöhen. Der Effekt

einer Entscheidungshilfe auf die informierte Entscheidung kann beurteilt werden. Auf Basis dieser Daten können Handlungsempfehlungen für verschiedene Akteure erarbeitet werden. So kann z.B. die Kooperationsgemeinschaft Mammographie die Ergebnisse des Projektes vielfältig verwenden. Auf der Basis des Projektes ist es möglich, die aktuell bestehenden, offiziellen Informationsmaterialen ggf. zu überarbeiten und an die Bedürfnisse der Frauen anzupassen, um eine informierte Entscheidung zu gewährleisten. Ein weiterer, wichtiger Nutzen des Projektes ergibt sich für Frauen bzw. Frauenorganisationen. Durch die Evaluation des Nutzens einer Entscheidungshilfe können bedarfsgerechte Materialien und Strukturen entwickelt werden, die den Anteil an voll informierten Frauen in allen Bevölkerungsgruppen erhöhen. Darüber hinaus liefert das Projekt auch Hinweise darauf, welche Aspekte jenseits von sachlichen Informationen für die Entscheidungsfindung und Teilnahme am Screening relevant sind.

Mit der in diesem Projekt entwickelten Entscheidungshilfe wird ein Werkzeug zur Entscheidungsfindung geschaffen, das in weiteren Studien zur informierten Entscheidung eingesetzt und in anderen Populationen evaluiert werden kann.

2.3.2 Mit welchen Risiken ist die Studie für die Versuchsteilnehmer verbunden?

Die Studienteilnehmerinnen werden keinen Risiken ausgesetzt.

2.3.2.1 Welcher Art sind die Risiken?

Für diesen Antrag nicht relevant.

2.3.2.2 Mit welcher Wahrscheinlichkeit ist zu erwarten, dass sich die Risiken realisieren? Wie sicher ist die Wahrscheinlichkeit abschätzbar?

Für diesen Antrag nicht relevant.

2.3.2.3 Wie ist das Risiko bestimmt worden? (Methode, Kriterien)

Für diesen Antrag nicht relevant.

2.3.3 Sind Kriterien festgelegt worden, bei deren Eintreten der gesamte Versuch abgebrochen werden soll? Wenn ja, welche?

Für diesen Antrag nicht relevant.

2.3.4 Ist das mögliche Risiko im Verhältnis zu dem zu erwartenden Nutzen Ihrer Ansicht nach vertretbar? Warum?

Für diesen Antrag nicht relevant.

2.4 Besondere Zulässigkeitsvoraussetzungen

2.4.1 Bei allen biomedizinischen Studien (auch bei Arzneimittel- und Medizinprodukteprüfungen)

Für diesen Antrag nicht relevant.

2.4.1.1 Sind alle geeigneten Vorprüfungen (z.B. bei Arzneimitteln: pharmakologisch-toxikologische Prüfung, bei Medizinprodukten: technische, biologische oder sonstige Sicherheitsprüfungen) durchgeführt und alle anderen, für Menschen ungefährlicheren Erprobungsmöglichkeiten ausgeschöpft?

Für diesen Antrag nicht relevant.

2.4.1.2 Wie sollen die Versuchsteilnehmer über Wesen, Bedeutung und Tragweite der Studie aufgeklärt werden? (Merkblatt beifügen)

Den potenziellen Studienteilnehmerinnen wird eine ausführliche Information über die Studie zugesendet (siehe Anlage).

2.4.1.3 Wie sollen die Versuchsteilnehmer ihre Einwilligung in die Teilnahme an der Studie erklären? (vorformulierte Erklärung beifügen)

Die Einwilligung erfolgt schriftlich. Die Frauen erhalten mit der Studieninformation eine Einverständniserklärung sowie einen frankierten Rückumschlag.

2.4.1.4 Sind, sofern einschlägig, die Bestimmungen der Röntgenverordnung und der Strahlenschutzverordnung eingehalten?

Für diesen Antrag nicht relevant.

2.4.2. Bei Studien an Minderjährigen (oder sonst nicht geschäftsfähigen Personen)

Es werden keine Minderjährigen befragt.

2.4.2.1 Warum kann die Studie nicht an Erwachsenen (voll Geschäftsfähigen) durchgeführt werden?

Für diesen Antrag nicht relevant.

2.4.2.2 Sind Aufklärung und Einwilligung der gesetzlichen Vertreter gewährleistet? (vorformulierte Erklärung beifügen!)

Für diesen Antrag nicht relevant.

2.4.2.3 Sind zusätzlich Aufklärung und Einwilligung der minderjährigen (nicht voll geschäftsfähigen) Versuchsteilnehmer gewährleistet, die selbst in der Lage sind, Wesen, Bedeutung und Tragweite des Versuchs einzusehen und ihren Willen danach zu bestimmen?

Für diesen Antrag nicht relevant.

2.4.3 Bei Arzneimittelprüfungen

Diese Studie beinhaltet keine Arzneimittelprüfung.

2.4.3.1 Welches Präparat soll geprüft werden? (bitte Angaben zu: Name, Beschreibung, Chemie der Substanz, Pharmakologie, Toxikologie, Pharmakokinetik)

Für diesen Antrag nicht relevant.

2.4.3.2 Ist das Präparat beim Bundesinstitut für Arzneimittel und Medizinprodukte zugelassen? Wenn nicht, sind die Unterlagen über die vorgesehene klinische Arzneimittelprüfung beim Bundesamt für Arzneimittel und Medizinprodukte vorgelegt?

Für diesen Antrag nicht relevant.

2.4.3.3 Ist der Projektleiter durch den Leiter der pharmakologisch-toxikologischen Prüfung über deren Ergebnisse informiert worden?

Für diesen Antrag nicht relevant.

2.4.3.4 Probandenversicherung

- **Ist zugunsten der Versuchsteilnehmer eine Versicherung abgeschlossen worden?**
- **Bei welcher Versicherungsgesellschaft und in welcher Höhe?**
- **Wie sollen die Versuchsteilnehmer über diese Versicherung, insbesondere über die den Versuchsteilnehmer im Versicherungsfall betreffenden Obliegenheiten, aufgeklärt werden?**

Für diesen Antrag nicht relevant.

2.4.4 Bei Medizinproduktversuchen

Diese Studie beinhaltet keine Medizinproduktversuche.

2.4.4.1 Was für ein Medizinprodukt soll geprüft werden (Name, Beschreibung, Eigenschaften)

Für diesen Antrag nicht relevant.

2.4.4.2 Hat das Medizinprodukt eine CE-Kennzeichnung für den beabsichtigten Gebrauch? Wenn nicht, sind die Unterlagen über die vorgesehene klinische Prüfung beim Bundesamt für Arzneimittel und Medizinprodukte vorgelegt?

Für diesen Antrag nicht relevant.

2.4.4.3 Ist der Leiter der klinischen Prüfung über die Ergebnisse der biologischen und technischen Sicherheitsprüfungen informiert worden?

Für diesen Antrag nicht relevant.

2.4.4.4 Probandenversicherung

- **Ist zugunsten der Versuchsteilnehmer eine Versicherung abgeschlossen worden?**
- **Bei welcher Versicherungsgesellschaft und in welcher Höhe?**
- **Wie sollen die Versuchsteilnehmer über diese Versicherung, insbesondere über**

die den Versuchsteilnehmer im Versicherungsfall betreffenden Obliegenheiten, aufgeklärt werden?

Für diesen Antrag nicht relevant.

3 Unterschrift des/ der Antragsteller(in/nen)

____

Ort, Datum Unterschrift

Prof. Dr. Petra Kolip
Universität Bielefeld
AG Prävention & Gesundheitsförderung

4 Einverständniserklärung des Direktors der Klinik, Abteilung, des Institutes

____

Ort, Datum Unterschrift

Prof. Dr. Petra Kolip
Universität Bielefeld
AG Prävention & Gesundheitsförderung

5 Anhang

5.1 Datenschutzrechtliche Stellungnahme des Datenschützers der Universität Bielefeld

5.2 Information für die Studienteilnehmerinnen

5.3 Einwilligungserklärung

5.4 Skizze der Entscheidungshilfe

5.5 Absichtserklärung Kooperationsprojekt
