# Supplementary material for: Does a decision aid improve informed choice in mammography screening? Results from a randomised controlled trial
Source: PLoS One. 2017 Dec 13;12(12):e0189148. doi: 10.1371/journal.pone.0189148 (PMC5728514; doi:10.1371/journal.pone.0189148)
Supplement: S2 File — (PDF) [file pone.0189148.s002.pdf]

Application for the review of a biomedical research project in humans by the ethics committee of the Medical Faculty of the Westfälische Wilhelms-Universität (University of Münster)

1 Formal information

1.1 Date of application

01.05.2013

1.2 Titel of the research project

"Development and Testing of a Decision Aid for Mammography Screening (EnTeMa)"

1.3 Details of the responsible project leader

1.3.1 Name, first name, academic degrees, official position

Kolip, Petra, Prof. Dr.

1.3.2 Address and telephone number, possibly fax (for inquiries regarding the application)

Universität Bielefeld
Fakultät für Gesundheitswissenschaften
AG für Prävention und Gesundheitsförderung
Universitätsstr. 25
33615 Bielefeld

Tel.: 0521/ 106 67273
Fax: 0521/ 106 6433
Email: petra.kolip@uni-bielefeld.de

1.3.3 Place and date of issuance of the licence to practise medicine

Not relevant for this application.

1.3.4 Place and date of issuance of the right to practice in the Federal Republic of Germany (only for foreigners)

Not relevant for this application.

1.3.5 For medicinal product tests: Can the project leader prove at least two years' experience in clinical testing of medicinal products?

Not relevant for this application.

1.4 Is the study carried out in collaboration with other investigators? (If applicable, details as for 1.3.1.-1.3.4.)

The study is carried out in cooperation with the project "Informed Choice of German and Turkish Women for Participation in the MSP (InEMa)". This study has already been approved. Project leaders are Jun.Prof. Dr. Jacob Spallek and Prof. Dr. Petra Kolip. A declaration of intent for the cooperation can be found in the appendix.

1.4.1 Name, first name, academic degrees, official position

Not relevant for this application.

1.4.2 Address and telephone number, possibly fax (for inquiries regarding the application)

Not relevant for this application.

1.4.3 Place and date of issuance of the licence to practise medicine

Not relevant for this application.

1.4.4 Place and date of issuance of the right to practice for the Federal Republic of Germany (only for foreigners)

Not relevant for this application.

1.3.5 For medicinal product tests: Can the project leader prove at least two years' experience in clinical testing of medicinal products?

Not relevant for this application.

1.5 Information on the Research Centre

1.5.1 Name of the institute, clinic, department or practice

Department for Prevention and Health Promotion, School of Public Health, Bielefeld University

1.5.2 Director/Head

Prof. Dr. Phil. Petra Kolip

1.5.3 Will the project be carried out in collaboration with other research centres (especially for multi-centre studies)? (If so, please name the research centre, its director/head, the responsible project leader, and the coordinator of the entire study.)

Not relevant for this application.

1.6 Information on funding

1.6.1 Please specify the initiator of the study (project leader/external sponsor, for example industry, others)

The study was initiated by Prof. Dr. Petra Kolip in cooperation with Jun.Prof. Dr. Jacob Spallek. Information on funding under 1.6.2.

1.6.2 Whom finances the project?

The project is financed by Bielefeld University.

2 Information on the content

2.1 Information on the research project

The project is conducted in cooperation with the project InEMa (Jun.Prof. Dr. Jacob Spallek, Prof. Dr. Petra Kolip; see appendix for declaration of intent). Certain passages, which apply to both studies, were adopted from the InEMa ethics application.

2.1.1 Aim of the study (Why is the study conducted? What is known? Preliminary studies? What shall be investigated?)

Decision aids are interventions to enable informed choices. They are already used in numerous health-relevant decision contexts. This study aims to clarify whether a decision is more informed when a decision aid about mammography screening has been used and whether demographic factors have an influence.

The following research questions will be investigated:
1. Does a decision aid increase the proportion of informed choices?
2. How does a decision aid affect the components of the informed choice (knowledge, values/attitude, intention, implementation of the intention)?
3. Does a decision aid increase the satisfaction with the decision?
4. Is the effect of the decision aid different depending on certain demographic factors, different outcome groups of the screening and the time spent with the decision aid?

In this study, the socio-economic status is recorded analogously to the study on the health of adults in Germany (DEGS) (Robert Koch Institute). This has the advantage that reference data on the socio-economic status are available.

2.1.2 Is it known whether studies with the same or a similar aim have been or are being carried out elsewhere?

The InEMa study assesses informed choice using the same measurement instrument but without providing a decision aid.

2.1.3 Procedure of the study? (Please describe the subject, the study procedure, the methodology, the dosage and application method, the type of application, etc., as precisely as possible, possibly with the aid of a schematic representation)

The instrument for measuring informed choice in the MSP has already been developed as part of the study InEMa. The decision aid is designed based on evidence-based data and based on existing decision aids for women under 50 and over 70 from other countries as well as decision aids from other screening contexts. The measuring instrument is used at two measurement times: shortly after receiving the invitation to the screening (T1) and about one month after the MSP appointment (T2). The decision aid is made available at the first measurement point.

The study includes the following arms:

1. Control group “Online-Survey”: Web-based questionnaire
2. Experimental group “Decision aid”: Web-based questionnaire, web-based decision aid

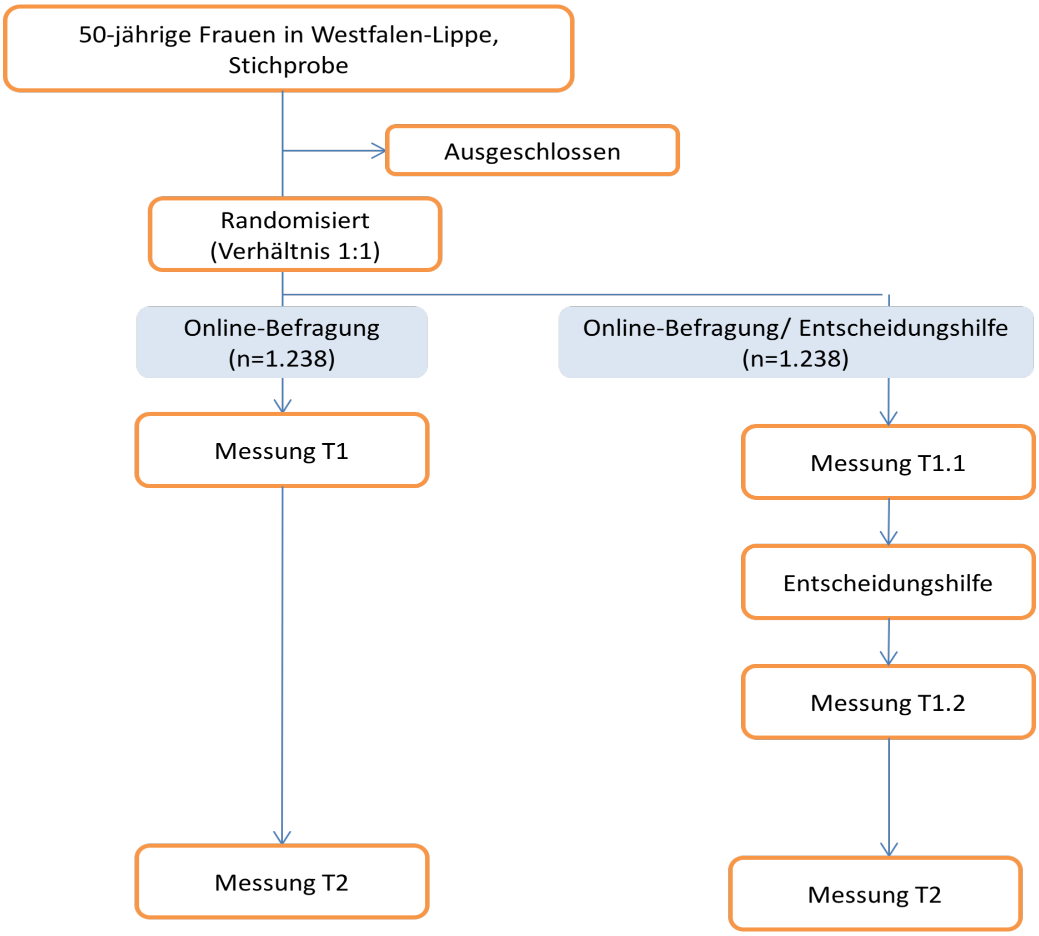

Figure 1: Overview of the study arms “online survey” and “decision aid”.

Both knowledge and attitude are assessed at both measurement times to evaluate the effect of decision aid. Intention is assessed at T1, self-reported retrospective participation at T2.

A decision aid for women who have been invited for the first time to the mammography screening program in Germany is being developed. This includes information on mammography screening, sample worksheets and a personal worksheet on personal values and the decision for (non-) participation. The women in the arm “decision aid” receive an information and decision-making tool in addition to the information brochure “Früherkennung von Brustkrebs. Was Sie darüber wissen sollten” (Kooperationsgemeinschaft Mammographie, Deutsches Krebsforschungszentrum - Krebsinformationsdienst, 2009). No other information is provided in the decision aid than in the brochure. Only the presentation and the interaction possibilities with the material differ. The probability of the different outcomes is presented as number of events per 200 women over 20 years supplemented by crowd figure pictograms. The decision aid will first be evaluated in a pre-test with 100 women.

Eligible women at the age of 50 in a screening region are surveyed online. The sample of study participants is drawn from the data of the registration offices in the administrative area of the Association of Statutory Health Insurance Physicians Westphalia-Lippe.

In the planned study region, the district of the Association of Statutory Health Insurance Physicians Westphalia-Lippe, there are 13 screening units. To ensure population coverage in the mammography screening program, invitations with a specific time and date are sent out based on data of the registration offices via so-called "Zentrale Stellen". The "Zentrale Stellen" generate invitation letters containing a specific appointment time and place from the address data of the eligible women. With the establishment of the "Zentrale Stellen", the personal data of the eligible women is separated from the service providers legally, spatially, and by staff. To ensure data protection, for this study samples of 50-year-old women are drawn independently by the respective registration offices. The data from the "Zentrale Stellen" is not used.

c) Choice of sample and sample size
The random sample for the study is drawn by the registration offices. All women aged 50 are included for the drawing of the sample as they are eligible for breast cancer screening within the MSP for the first time. Women who have already had a breast cancer diagnosis are excluded from the analysis because they, as diseased women, are not the target group of the MSP.

In each arm of the study 1,238 women are to be included. A change in the proportion of informed choices as well as a knowledge increase are expected with an effect size of .1. The required total sample size for a Type I error rate of 5% and a Type II error rate of 20% is N = 2,474. Since a response rate of 10% is expected, 24,740 women will be invited to participate in the study. The recruitment takes place in several waves, depending on how quickly the required number of participants is reached.

d) Recruitment/feasibility
Detailed information about the study will be sent by mail to the women. The women receive together with the study information a declaration of consent with stamped return envelope as well as the link to the first questionnaire and, if applicable, the decision aid.

e) Intervention
In the arm "decision aid", a decision aid is offered which contains the same information as the brochure "Früherkennung von Brustkrebs. Was Sie darüber wissen sollten" (Kooperationsgemeinschaft Mammographie & Deutsches Krebsforschungszentrum - Krebsinformationsdienst, 2009).

f) Secondary data analysis
Secondary data analyses are planned to compare this study with the cooperation project InEMa. It will be particularly interesting to see whether there are differences in the response rate due to the survey mode (paper-and-pencil vs. online).

g) Control of potential confounders
For the survey, a random sample of the invited women is drawn based on the data of the registration offices. The selected design is a randomized controlled trial.

A potential confounder for the results of the study are previous screening experiences of the women. Therefore, for the study only women aged 50 are surveyed, who are invited for the

first time to the mammography screening. In addition, prior knowledge on breast cancer screening and breast cancer diagnosis are assessed.

A possible problem with using an online survey for a population sample is a low and selective rate of participation. The statistical analyses can be adjusted for various possible confounders by applying stratified and multivariate methods.

h) Statistical analysis/evaluation
Descriptive analyses are conducted to describe the study population and the individual social groups. To compare the arm "decision aid" with the arm "online survey", statistical methods for group comparison are used (e.g., ANOVA, chi^2-test). Multivariate relationships are tested using linear and logistic regression.

2.1.3.1 In which form is the study conducted? Is it

- A pharmacological, clinical-pharmacological, clinical or other biomedical study?

- An open or single or double blinded study?

- A randomised study?

- A diagnostic, therapeutic, tolerability or exclusively scientific study?

This study is a randomized controlled trial. The sample is randomised either to the arm "online survey" or the arm "decision aid".

2.1.3.2 By whom is the trial participant medically supervised before, during and after the study? (Coordination with the GP, control of other medications?)

No medical care is required.

2.1.3.3 Duration of the study

The study is planned for 3 years. In the context of the InEMa project, the study preparation (especially literature research and questionnaire development) began as early as October 2012. The study ends in September 2015.

2.1.3.4 Are interim results evaluated to identify a trend? What consequences for the participants are drawn according to which criteria?

The decision aid will be published after its development. A change of the research project during the implementation is not planned since after a careful pre-test and considering the sample size this would not be useful. No negative impact on the participants is expected as the decision aid does not contain any information other than the above-mentioned brochure which the women receive anyway together with the invitation to the MSP.

2.1.3.5 What kind of documentation is provided? (If applicable, include a copy of the documentation forms)

Not relevant for this application.

2.1.3.6 Is the collaboration of a statistician planned and which statistical methods are to be used?

The above-described statistical methods are employed. The collaboration of a statistician is not planned, since the study researchers themselves have experience in the statistical evaluation of RCTs.

2.1.3.7 Are medical confidentiality and data protection regulations observed?

Data handling is in accordance with the data protection regulations of the state of North Rhine-Westphalia. A positive votum of the data protection officer of Bielefeld University is provided (see Appendix).

2.1.4 Shall the participants be paid a compensation (compensation for expenses etc.)? In which amount?

The study participants receive no compensation for expenses and no incentives.

2.1.5 Shall the investigators involved be paid a remuneration? In which amount?

Not relevant for this application.

2.2 Information on the trial participants

2.2.1 Number (for comparative studies, please indicate division into groups)

A pre-test of the decision aid will be conducted with 100 women.

In the actual study, a total of 2,476 women are to be included, 1,238 each in the control and experimental group.

2.2.2 Age and gender (please indicate the age of the participants and the upper and lower limits as exclusion criteria)

Only women aged 50 are surveyed as they receive an invitation to mammography-screening for the first time.

2.2.3 Status: Are the trial participants
a) Healthy persons?
b) Pregnant or breastfeeding women?
c) Diseased persons? (Please indicate the disease and the stage)
d) Inpatients or ambulatory patients?
e) Persons suffering from other diseases? (In particular, mental illnesses, which give rise to doubts about the legal right capacity and ability?)

The study participants are not selected according to their health status to participate in the study.

2.2.4 What other inclusion criteria (for example, allowable accompanying medication) are planned?

None.

2.2.5 What other exclusion criteria (for example, advanced renal or hepatic impairment, forbidden accompanying medication, etc.) are planned?

None.

2.2.6 Shall persons participate, who are under custody in an institution according to a judicial or official decree?

No.

2.2.7 Shall persons participate, who are already enrolled in other research projects (if applicable, when and how often)?

No.

2.3 Information on the benefit-risk ratio

2.3.1 Which benefit is to be expected from the study results

a) for the trial participants?

For the participants in the experimental group a higher proportion of informed choices is to be expected.

b) for medical science?

No direct benefit.

c) for science (e.g., results not for immediate therapeutic purposes)?

The overall aim of this study is to contribute to increasing the proportion of informed choices for or against participation in the MSP. The effect of a decision aid on informed choice can be assessed. Based on these data, recommendations for different stakeholders can be developed. Thus, e.g. the Kooperationsgemeinschaft Mammographie may use the results of the project versatilely. Based on the project, it is possible to revise the existing official information materials and adapt them to the needs of the women to ensure an informed choice.

Another important benefit of the project is for women or respectively women's organizations. Through the evaluation of the benefit of a decision aid, it may be possible to develop needs-based materials and structures that increase the proportion of fully informed women in all population groups. In addition, the project also provides indications as to which aspects, beyond factual information, are relevant for decision-making and screening participation.

The decision aid developed in this project is a decision-making tool that can be used in further studies on informed choice and can be evaluated in other populations.

2.3.2 What are the risks for the trial participants?

The study participants are not exposed to any risks.

2.3.2.1 What kind of risks are there?

Not relevant for this application.

2.3.2.2 With which probability can the risks be expected to occur? How reliably can the probability be estimated?

Not relevant for this application.

2.3.2.3 How has the risk been determined? (Method, criteria)

Not relevant for this application.

2.3.3 Have criteria been determined in which case the entire trial should be terminated? If yes, which?

Not relevant for this application.

2.3.4 Is the possible risk in relation to the expected benefit justifiable in your view? Why?

Not relevant for this application.

2.4 Special admissibility requirements

2.4.1 For all biomedical studies (also for medicinal product and medical device tests)

Not relevant for this application.

2.4.1.1 Have all suitable preliminary tests (for example for medicinal products: pharmacological and toxicological tests, for medical devices: technical, biological or other safety tests) been conducted and have all other testing possibilities being more harmless to humans been exhausted?

Not relevant for this application.

2.4.1.2 How shall the trial participants be informed about the nature, significance and scope of the study? (include leaflet)

A detailed information about the study will be sent to potential study participants (see appendix).

2.4.1.3 How shall the participants declare their consent to participate in this study? (include pre-formulated declaration)

The consent is given in writing. The women receive with the study information a declaration of consent as well as a stamped return envelope.

2.4.1.4 Are the provisions of the X-ray Ordinance and of the Radiation Protection Ordinance, if applicable, observed?

Not relevant for this application.

2.4.2. For studies with minors (or other persons not possessing legal right capacity and ability)

No minors are surveyed.

2.4.2.1 Why can the study not be conducted with adults (possessing legal right capacity and ability)?

Not relevant for this application.

2.4.2.2 Are the information and consent of the legal representatives ensured? (include pre-formulated declaration!)

Not relevant for this application.

2.4.2.3 Are additional information and consent of the minor (not possessing legal right capacity and ability) participants ensured, who are themselves in a position to determine the nature, meaning and scope of the trial and to determine their will accordingly?

Not relevant for this application.

2.4.3 For medicinal product tests

This study does not include any medicinal product tests.

2.4.3.1 Which medicinal product shall be tested? (Please specify: name, description, chemical substance, pharmacology, toxicology, pharmacokinetics)

Not relevant for this application.

2.4.3.2 Is the medicinal product licensed by the Federal Institute for Drugs and Medical Devices? If not, have the documents on the planned clinical trial been submitted to the Federal Institute for Drugs and Medical Devices?

Not relevant for this application.

2.4.3.3 Has the project leader been informed about the results of the pharmacological and toxicological tests by the head of pharmacological and toxological tests?

Not relevant for this application.

2.4.3.4 Insurance for the participants of the clinical trial
- **Has an insurance contract been entered to the benefit of the trial participants?**
- **With which insurance company and for which amount?**
- **How shall the trial participants be informed about this insurance, especially about obligations concerning the trial participants?**

Not relevant for this application.

2.4.4 For medical device tests

This study does not include any medical device tests.

2.4.4.1 What kind of medical device shall be tested (name, description, characteristics)

Not relevant for this application.

2.4.4.2
Does the medical device have a CE marking for the intended use? If not, have the documents on the planned clinical trial been submitted to the Federal Institute for Drugs and Medical Devices?

Not relevant for this application.

2.4.4.3 Has the head of the clinical trial been informed about the results of the biological and technical safety tests?

Not relevant for this application.

2.4.4.4 Insurance for the participants of the clinical trial

- **Has an insurance contract been entered to the benefit of the trial participants?**
- **With which insurance company and for which amount?**
- **How shall the trial participants be informed about this insurance, especially about obligations concerning the trial participants?**

Not relevant for this application.

3 Signature of the applicant(s)

____

Place, Date Signature

Prof. Dr. Petra Kolip
Universität Bielefeld
AG Prävention & Gesundheitsförderung

4 Consent of the director of the clinic, department, institute

____

Place, Date Signature

Prof. Dr. Petra Kolip
Universität Bielefeld
AG Prävention & Gesundheitsförderung

5 Appendix

5.1 Data protection statement of the data protection officer of Bielefeld University

5.2 Information for study participants

5.3 Consent form

5.4 Draft of the decision aid

5.5 Declaration of intent for the cooperation project
